# The Effect of an Infant Formula Supplemented with AA and DHA on Fatty Acid Levels of Infants with Different FADS Genotypes: The COGNIS Study

**DOI:** 10.3390/nu11030602

**Published:** 2019-03-12

**Authors:** Isabel Salas Lorenzo, Aida M. Chisaguano Tonato, Andrea de la Garza Puentes, Ana Nieto, Florian Herrmann, Estefanía Dieguez, Ana I. Castellote, M. Carmen López-Sabater, Maria Rodríguez-Palmero, Cristina Campoy

**Affiliations:** 1 Department of Nutrition, Food Sciences and Gastronomy, Faculty of Pharmacy and Food Sciences, University of Barcelona, Av. Joan XXIII 27-31, E-08028 Barcelona, Spain; salas.lorenzo.i@gmail.com (I.S.L.); aicastellote@ub.edu (A.I.C.); 2 Institut de Recerca en Nutrició i Seguretat Alimentària de la UB (INSA-UB), 08921 Barcelona, Spain; 3 Nutrition, Faculty of Health Sciences, University of San Francisco de Quito, Quito 170157, Ecuador; achisaguano@usfq.edu.ec; 4 Parc Sanitari Sant Joan de Déu, Fundació Sant Joan de Déu, Institut de Recerca Sant Joan de Déu, 08830 Sant Boi de Llobregat, Spain; 5 Centre of Excellence for Paediatric Research EURISTIKOS, University of Granada, 18071 Granada, Spain; ananietoruiz@gmail.com (A.N.); herrmann@florian-herrmann.de (F.H.); estefaniadieguez@ugr.es (E.D.); ccampoy@ugr.es (C.C.); 6 Department of Paediatrics, University of Granada, 18071 Granada, Spain; 7 CIBER Physiopathology of Obesity and Nutrition CIBERobn, Institute of Health Carlos III, 28029 Madrid, Spain; 8 Basic Research Department. Ordesa Laboratories, 08830 Barcelona, Spain; Maria.Rodriguez@ordesa.es; 9 CIBER Epidemiology and Public Health CIBEResp, Institute of Health Carlos III, 28029 Madrid, Spain

**Keywords:** fatty acids, omega 6, omega 3, breast milk, infant formula, fatty acid desaturases, early life nutrition, control formula, intervention formula, exclusive breastfeeding

## Abstract

Polymorphisms in the fatty acid desaturase (FADS) genes influence the arachidonic (AA) and docosahexaenoic (DHA) acid concentrations (crucial in early life). Infants with specific genotypes may require different amounts of these fatty acids (FAs) to maintain an adequate status. The aim of this study was to determine the effect of an infant formula supplemented with AA and DHA on FAs of infants with different FADS genotypes. In total, 176 infants from the COGNIS study were randomly allocated to the Standard Formula (SF; n = 61) or the Experimental Formula (EF; n = 70) group, the latter supplemented with AA and DHA. Breastfed infants were added as a reference group (BF; n = 45). FAs and FADS polymorphisms were analyzed from cheek cells collected at 3 months of age. FADS minor allele carriership in formula fed infants, especially those supplemented, was associated with a declined desaturase activity and lower AA and DHA levels. Breastfed infants were not affected, possibly to the high content of AA and DHA in breast milk. The supplementation increased AA and DHA levels, but mostly in major allele carriers. In conclusion, infant FADS genotype could contribute to narrow the gap of AA and DHA concentrations between breastfed and formula fed infants.

## 1. Introduction

Long chain polyunsaturated fatty acids (LCPUFA) have an important role in the immune system regulation, blood clots, neurotransmitters, cholesterol metabolism, and in the structure of membrane phospholipids in the brain and the retina [1]. Attention has been devoted especially to arachidonic acid (AA) and docosahexaenoic acid (DHA), due to their key role for optimal health, cognition and development during fetal and early postnatal life [2]. Breastmilk is usually the only external source of AA and DHA for infants during the first months of life [3,4]. When breastfeeding is not possible, infants require breast milk substitutes [5], which are usually supplemented with nutrients to match the breast milk content [6]. However, there is still debate and different opinions in regards of DHA and AA supplementation in infant formulas. The European Food Safety Authority (EFSA) [7] and the Commission Delegated Regulation (EU) 2016/127 [8] have proposed DHA supplementation as mandatory for infant formulas, while no minimum amount of AA was determined to be necessary, setting AA supplementation as an optional ingredient. However, it has been observed that when infants receive both AA and DHA supplementation they have better outcomes in cognitive performance than receiving DHA alone [9]. Other authors have even found that DHA alone did not influenced cognitive development at all [10]. Therefore, whether DHA should be supplemented alone or with AA remains controversial. Additionally, there is no agreed specific dose for supplementation, and there is little evidence of the long-term effect.

LCPUFAs can also be endogenously synthesized from the essential fatty acids (FAs): linoleic acid (LA) and alpha-linolenic acid (ALA). LCPUFA synthesis requires desaturation and elongation reactions. D6 and D5 desaturases (D6D and D5D) are two key enzymes that catalyze the synthesis by introducing *cis* double bonds at specific positions. Fatty acid desaturase genes FADS1 and FADS2 encode D5D and D6D, respectively, making them a rate limiting factor in LCPUFA conversion [11]. However, the endogenous synthesis of AA and DHA from their precursors is limited in humans as Demmelmair et al. observed in women that only 1.2% of the AA is directly derived from LA intake [12]. Likewise, both blood and tissue levels of PUFAs are influenced to a large extent by genetic heritability [13]. There are many studies showing the major impact of gene variants of the FADS gene cluster on the FA composition of blood, tissues and human milk [14,15,16]. For instance, single nucleotide polymorphisms (SNPs) in the FADS gene modulate the capacity for endogenous synthesis of LCPUFAs by compromising the desaturase activity of the involved enzymes [17,18,19,20,21]. It has been observed that up to 28% of variation of AA blood levels is due to FADS genetic variants, while LA is affected in 9% [13]. Furthermore, Baylin et al. observed that an impaired desaturase activity may induce an unbalanced proportion of n3 (e.g., the FADS2 deletion could prevent the conversion of the precursor ALA into LCPUFAs) [22]. Likewise, Schaeffer et al. observed that variants in the FADS1 and FADS2 genes showed strong associations with levels of n6 (e.g., LA, gamma-linolenic acid (GLA), dihomo-gamma-linolenic acid (DGLA), AA, and adrenic acid (AdA)), and n3 FAs (ALA, eicosapentaenoic acid (EPA), docosapentaenoic acid (DPA) [13], and DHA [23]). It has also been observed that variants in the FADS cluster can also influence total LDL, and DHL cholesterol, triglycerides, phospholipids, C-reactive protein, proinflamatory eicosanoids and cardiovascular disease endpoints [24,25].

Even though there is wide evidence of the effect of FADS genetic variants in FA concentrations of different biological tissues, there is little evidence for infants during their first year of life [19,26,27,28], which is a critical period of early life programming in which LCPUFAs play an important role [29]. Studying this could identify vulnerable groups in the pediatric population and contribute to the refinement of current recommendations and legislations in regards to infant AA and DHA supplementation. Given the low rates of exclusive breastfeeding in Europe, and while efforts are still being made to promote it, it is important to secure an appropriate source of LCPUFAs for infants who are not breastfed to promote their development. Therefore, the aim of this study was to determine the effect of an infant formula supplemented with AA and DHA on fatty acid levels of three-month-old infants with different FADS genotypes.

## 2. Materials and Methods

### 2.1. Ethics Statement

This study was carried out in accordance with the ethical standards established by the Declaration of Helsinki (2004), the Good Clinical Practice recommendations of the EEC (document 111/3976/88 July 1990) and the current Spanish legislation governing clinical research in humans (Royal Decree 561/1993 on clinical trials). In addition, the study was approved by the San Cecilio University Hospital Ethics Committee and the Faculty of Medicine at the University of Granada.

### 2.2. Study Population and Design

**Figure 1:**
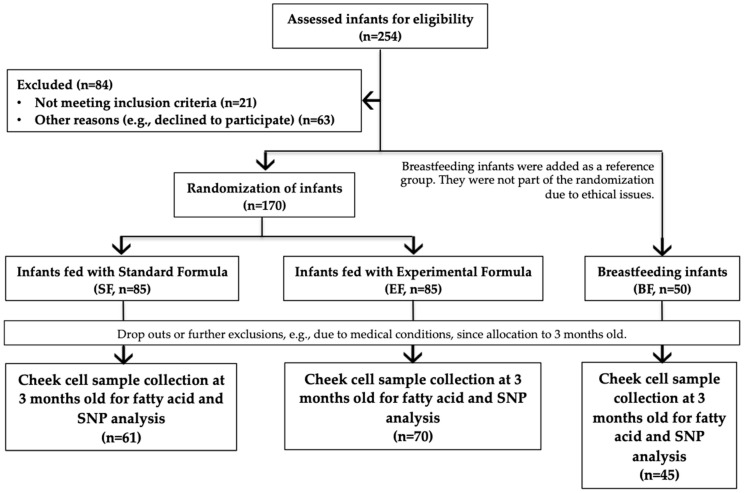
Participants in the COGNIS study and classification following type of feeding.

We analyzed 176 infants from the total of 220 participants in the COGNIS study (A Neurocognitive and Immunological Study of a New Formula for Healthy Infants), which is an interventional, randomized, and double-blinded study registered at www.ClinicalTrials.gov (NCT02094547). Full-term infants were recruited at the University Hospitals (Clinical San Cecilio and Mother-Infant Hospital) in the city of Granada (Spain), where samples and data were also collected. Each parent or legal guardian signed a written informed consent before the recruitment. To be eligible for enrolment of COGNIS study, infants had to meet the following inclusion criteria: 0–2 month old full-term infants, adequate birth weight for gestational age, normal Apgar score, umbilical pH ≥ 7.10 (normal range), availability to continue throughout the entire study period, signed informed consent, and for infants in the SF and EF groups, a maximum of 30 days of exclusive breastfeeding was considered and a minimum of 70% of infant formula consumption was required afterwards. Participants were excluded if they were participating in other studies, if they had nervous system or gastrointestinal disorders, and if the mother had a disease history or had received harmful drug treatment during pregnancy. Questionnaires and medical records were used to obtain maternal characteristics, including maternal age, gestational age, pre-pregnancy BMI, pre-pregnancy weight, smoking status during pregnancy, educational level, and Edinburgh scale. Likewise, infant characteristics such as gender, birth weight, birth length, and birth head circumference were obtained. The study design and information of the COGNIS participants are given in Figure 1.

After inclusion, infants were randomly allocated to the Standard Formula (SF) or the Experimental Formula (EF) group. Later, a third group was added with infants who were Exclusively Breastfed (BF) for at least 2 months to function as the control group.

After infant randomization, the research team decided to withdraw from the trial those infants who met the following criteria: Infants fed with infant formulas unrelated to COGNIS study, breastfed infants with formula intake >25% before 6 months, formula fed infants with human milk intakes higher than 25% beyond the 3rd month of life, any adverse event that could interfere with study follow-up, cow’s milk protein allergy/intolerance or lactose intolerance, infant formula intake rejection or neurological disorder.

All infants from COGNIS study were followed up at 2, 3, 6, 12, 18 months of life and 2.5 years of age. During the follow-up visits, and depending on the subject age, different assessment procedures and data collection were carried out; however, those will be explored elsewhere. Nevertheless, for the purpose to obtain a better control of records avoiding possible twists on data due to factors that involved other time lines, such as mixed feeding in the 1st month of life or initiation of complementary feeding at 6 months, this study only takes into account infants from the second visit (3 months of age).

### 2.3. Formulas

Infant formulas from SF and EF were based on cow’s milk and were provided by ORDESA Laboratories, S.L., Barcelona, Spain. The experimental formula was characterized by the presence of LCPUFAs AA (*Mortierella alpine*) and DHA (fish oil), milk fat globule membrane (MFGM) components {10% of total protein content (wt:wt}, symbiotics, gangliosides, nucleotides and sialic acid (Nutriexpert^®^ factor). Fat blend, OMEGA FATS (palm, palm-kemel, rapeseed, sunflower, oleic sunflower fatty acids) and BETAPOL (Palm, palm-kemel, sunflower and rapeseed oils) were present in both formulas. For more information regarding the lipid profile, consult the Supplementary Materials (Table S2). Additionally, both formulas followed the guidelines of the Committee on Nutrition of the European Society for Pediatric Gastroenterology, Hepatology and Nutrition (ESPGHAN), and the international and national recommendations for the composition of infant formulas. Nutritional composition of infant formulas is shown in Table 1.

### 2.4. Cheek Cell Sample Collection

Cheek cell samples were collected at 3 months of age to analyze FAs and genotype FADS SNPs. Samples were collected 1 h after feeding by scraping the inside of the cheeks with a Rovers^®^ EndoCervex-Brush^®^. The tip of the brush was transferred and jolted in a cryotube with distilled water, shaking the tip before removing the brush. After centrifugation, the supernatant was carefully discarded. The cell pellets were stored at −80 °C until analysis.

### 2.5. Cheek Cell Fatty Acid Analysis

A modified version of the method described by de la Garza et al. [30] was used to analyze FAs from the glycerophospholipid fraction. Methanol with butylated hydroxytoluene (BHT) was used for lipid extraction. FA reactions with sodium methylate in methanol (25 wt% in methanol) and boron trifluoride methanol solution (14% *v*/*v*) were used to obtain FA methyl esters (FAMEs). Next, the FAs were separated by rapid gas chromatography following the method developed by Bondia et al. [31]. The system consisted of a Shimadzu GC-2010 gas chromatograph (Kyoto, Japan) equipped with a “split-splitless” injector, an automatic injector with AOC-20i-AOC-20s sampler and a flame ionization detector (FID). The separation of the methyl esters from the FAs was carried out with a fast capillary column of fused silica VF-23ms (10 m × 0,10 mm internal diameter, 0,10 μm film thickness) coated with a stationary phase 100% cyanopropyl-phenyl-methyl-polysiloxane of varian (Palo Alto, CA, USA). The methyl esters of the FAs were identified by comparison with the retention times of standards, FAME-37 and PUFA-2 animal. Quantification was done by normalization, expressing the results in relative amounts (percentage). Enzyme activities were estimated as product:precursor indexes of individual FAs as follows: GLA:LA and DGLA:LA indexes for D6D enzyme activity, and the AA:DGLA index for D5D enzyme activity. Additionally, AA:LA and eicosapentaenoic acid (EPA):ALA indexes were analyzed.

### 2.6. SNP Selection and Genotyping

SNPs within the FADS gene were selected if they were documented in previous studies for comparison purposes [19,27,32,33,34,35,36,37,38] and if their minor allele frequency (MAF) was higher than 10%. DNA material was extracted from infant cheek cells. FADS1 (rs174537, rs174545, rs174546, rs174548, and rs174553) and FADS2 (rs1535, rs174570, and rs2072114) SNPs were genotyped from 2.5 µl of DNA mixed with 2.5 µL of 2X TaqMan^®^ OpenArray^®^ Genotyping Master Mix. Analysis was then performed with 4 µL of the mixture in a microplate using the TaqMan^®^ OpenArray^®^ genotyping technology. Analyses were carried out at the *Autonomous University of Barcelona* (UAB) using the QuantStudio 12 k Flex^®^ instrument (ThermoFisher) and the corresponding OpenArray^®^ SNP Genotyping Analysis software.

### 2.7. Statistical Analysis

SPSS statistical software package for Windows (version 20.0; SPSS Inc., Chicago, IL, USA) was used to perform the statistical analyses. Data were tested for normality using the Kolmogorov-Smirnov test and non-normal data were log transformed. This exploratory study evaluated the associations between SNPs and PUFAs within the study groups using a linear regression analysis. We decided to analyze each SNP individually to provide more evidence about their effects on LCPUFAs levels in the first stage of life, given the lack of literacy in this period of life. Heterozygotes and minor allele homozygotes were analyzed together as one group to improve sample size. However, this codification implies an additive and dominant model. SNPs were studied as a numeric variable by coding them according to the minor allele count; 0 for major homozygotes and 1 for heterozygotes and minor allele homozygotes. We also tested the analyses with the three allele groups and confirmed that the results showed the same tendency. The Hardy-Weinberg equilibrium and genotype distribution were analyzed with the x^2^-test (Supplementary Materials Table S1). We used a multivariate general linear model (GLM) to compare FA levels (mean ± standard deviation) between the study groups and according to FADS genotype. FAs were expressed as the percentage of total FAs. The analyses were corrected for potential confounders such as maternal characteristics (pre-pregnancy body mass index, age, education and smoking status), and gender of the child. The *p*-value cut-off has been reconsidered and changed according to Bonferroni correction (0.05/8 SNPs × 3 groups = 24) and assuming a moderate correlation of 30% between SNPs. The significance cut-off values resulted at <0.005 and this has been applied to each trait.

## 3. Results

### 3.1. Sample Characteristics

The population has the characteristics shown in Table 2. Mothers from the BF group were more likely to have a higher level of education and were older than mothers from the EF group. The EF group had more male infants than the BF group. No differences were observed in infant anthropometric data.

### 3.2. Associations of FADS SNPs with Fatty Acids

Table 3 shows nominal and significant associations between PUFAs and FADS minor alleles after adjusting for maternal age, maternal education, maternal smoking habit, and sex of infant.

The most significant associations (*p* < 0.005) were found in the EF group, where minor allele carriership of rs174537 was negatively associated with LA, AA and DHA (βc −0.376, −0.440, and −0.415, respectively), and FADS minor allele carriership of rs2072114 was negatively associated with AA and the AA:LA index (βc −0.522 and −0.450, respectively).

Within the SF group, nominal negative associations were found after correcting for confounders, while the BF group showed none.

The complete analysis can be found in Supplementary Materials Table S3.

### 3.3. Fatty Acid Comparison by FADS Genotype among Feeding Practice Groups

LCPUFA levels were statistically different among the feeding practice groups when classifying infants by FADS genotype (Table 4). Among major homozygotes, both the BF and EF groups had a higher AA level than the SF group, but when infants carried minor alleles, those with breastfeeding showed a higher AA level than both the EF and SF groups. DHA levels also exhibited differences. Among major homozygotes, infants in the BF and EF groups showed a higher DHA level than infants in the SF group. On the other hand, among minor allele carriers, the EF group presented a higher DHA level than the SF group, but the BF group had the highest DHA level of all. The complete analysis can be found in Supplementary Materials Table S4.

## 4. Discussion

The present study analyzed the influence of an infant formula, supplemented with AA + DHA, on LCPUFA levels in infants with different FADS genotype. A number of studies have investigated the association between variants in the FADS gene cluster and FA levels in human tissue [13,14,22,23,24]; however, little information is available on the neonatal population. Other studies have analyzed the effect of LCPUFA supplementation in early life [9,39,40,41], but no association with FADS SNPs has been investigated whatsoever. Our study contributes to generating new evidence in this matter.

FADS minor alleles have shown to decrease the desaturase activity [13,16,17,42,43,44,45,46], compromising LCPUFA production. Some authors have demonstrated that FADS SNPs lower proportions of GLA, AA, and EPA, and accumulate the LCPUFA precursors LA and ALA [11,46,47]. Others have demonstrated that FADS minor allele carriers exhibit lower AA:DGLA and EPA:ALA indexes [48,49]. As mentioned before, some studies include indexes of AA to LA, as well as EPA and DHA to ALA in order to use them as markers of the activity of fatty acids desaturation mediated by the enzymes D5D and D6D, respectively; in our study, we use GLA:AA and DGLA:LA to indicate D6D activity, whereas D5D activity is reflected by the ratio of AA to DGLA, and we consider it relevant to record if an infant formula supplemented with LCPUFA can influence the enzymatic activity of infants compared with non-supplemented, to allow a possible comparison for future studies. Moreover, it has been established that the AA is the most severely affected FA [13]. In our study, infants from the BF group carrying FADS minor alleles were not associated with FAs, which suggests that desaturase activity is not affected by FADS genotype when infants are exclusively breastfed.

However, infants carrying FADS minor alleles in the EF and SF groups were associated with decreased D5D and D6D activities, which is in line with the previous studies [46,50,51,52]. The most affected group was the EF, where infants carrying minor alleles of rs173547 (FADS1) had decreased levels of LA, AA and DHA, and minor allele carriers of rs2072114 (FADS2) were negatively associated with AA and the D5D and D6D desaturases activity (AA:LA index) (*p* < 0.005). Accordingly, the nominal associations (*p* < 0.05) were also inclined to decrease the desaturase activities when infants presented FADS minor alleles. In the EF group this was observed with n6 PUFAs (LA, DGLA, AA, AdA, DPAn6, AA:DGLA), EPA and DHA. Moreover, in the SF group the SNP rs174570 (FADS2) stood out by associating with decreased levels of AA, GLA:LA, DGLA:LA, AA:LA and EPA:ALA. These results suggest that the infants without AA and DHA supplementation were the least affected in terms of FA levels by FADS genetic variants. To make a proper interpretation of the results, it is of interest to take into account the different effect size among the study groups. For instance, the strongest association was found within the EF group, where minor allele carriers of rs2072114 showed the highest negative association with AA (βc −0.522, *p* < 0.001).

In our study, AA and DHA levels, in spite of the genotype, showed a gradient of SF < EF < BF. More specifically, among major homozygotes of FADS SNPs (except rs174570), AA and DHA concentrations were closer between the EF and the BF groups, and higher compared to SF infants, which shows the expected effect of the infant formula supplementation. Nevertheless, when carrying minor alleles, the EF group did not reach similar levels to the BF infants. In summary, Table 4 should be interpreted with caution due to effect size differences among study groups (e.g., the difference between the FA means of EF and BF groups is narrower when infants carry major homozygotes, and these differences increase among minor allele carriers).

This study exposes minor allele carriers as a potential vulnerable group since the same supplementation might not be enough for them, especially in the case of AA that showed the same levels than the SF group, whereas the DHA was at least higher than them.

Increasing the supplemented dose of preformed LCPUFAs may compensate for the effect of FADS minor alleles. According to Miklavcic et al., increasing the formula supplementation to AA 34 mg/100 kcal and DHA 17 mg/100 kcal prevents the reduction in AA by minor alleles [53]. However, in our study, the EF group received a supplementation of 23.2 mg/100 kcal of AA and 15.5 mg/100 kcal of DHA, which could be a reason for their suggested high SNP influence. As for the SF infants, their AA and DHA levels were almost unaffected by minor alleles. Since previous evidence suggested that high supplementation levels of AA and DHA could lessen the influence of FADS SNPs on LCPUFA levels, we somewhat expected a gradient effect of SNPs of SF > EF > BF. However, this expectation was not determined since the SF group was not supplemented at all. The gradient resulted in EF > SF > BF. It is interesting how the LCPUFA levels of the SF group were less influenced by the FADS SNPs than the EF group. We could speculate some kind of protection effect (e.g., a more effective synthesis in the absence of preformed AA and DHA intake [54]) against FADS SNPs when infants have low LCPUFA levels. It is important to remember that, even though the EF group was more influenced by FADS SNPs than the SF group, infants from the EF group still presented higher AA and DHA levels. Corresponding to the theory of the higher the LCPUFA supply the less SNP influence, AA and DHA levels of BF infants were not perceptibly affected by genotype, possibly because they had the highest LCPUFA concentrations related to the high content in breast milk. One should consider that mean fat content of human milk may vary considerably between individuals as well as between study populations from affluent or developing countries [55]. Maternal factors, such as diet and weight gain during pregnancy, and sampling procedures have distinct impact on fat levels [56]. Additionally, DHA levels in breast milk are quite sensitive to maternal diet [4] and maternal FADS genotypes are associated with breast-milk AA concentration and this might therefore influence the supply of breast milk FAs [42].

Several authors have demonstrated that FADS SNPs affect FA levels and are associated with health conditions [13,39,57,58,59,60,61,62]. The major contribution of our study is providing evidence in infants below 6 months of age, identifying a potential vulnerable group of infants who could benefit from more personalized nutrition, and contributing to defining the ideal AA and DHA supplementation of infant formulas. This line of research merits further attention because early life nutrient exposure can program future health. We acknowledge some limitations, such as the lack of information on maternal dietary intake and supplementation, and the relatively small sample size of our population. We should also take into consideration the estimation of effect size to interpret the influence of SNPs on LCPUFA levels according to the study group. Now that we have evidence for each individual SNP, it is of interest to perform a FADS haplotype analysis to compare the results. The strengths of our study include the double-blinded cohort study design and the participation of three infant groups with different feeding practices. It is important to mention that the infant formulas were created previous to the implementation of the Commission Delegated Regulation (EU) 2016/127 [8] when DHA supplementation was not mandatory.

## 5. Conclusions

In conclusion, formula fed infants with FADS minor alleles, especially those with AA and DHA supplementation, were associated with decreased desaturase activity and lower AA and DHA levels. Breastfed infants were not affected, possibly due to the high LCPUFA content in breast milk. The AA and DHA supplementation of the infant formula provided the infants carrying major allele homozygotes with closer levels to those obtained with breastfeeding. This exposes minor alleles as a potential factor of vulnerability since the same supplementation might not be enough for them. Considering infant FADS genotype to meet the individual needs could contribute to narrow the gap of AA and DHA concentrations between breastfed and formula fed infants. A new LCPUFA supplementation dose should be explored to determine if an increased supplementation of AA and DHA might prevent the reduction of these FAs observed in the presence of FADS minor alleles. However, when breastfeeding is not possible, supplemented formulas should be considered as the second choice since they provide better AA and DHA concentrations compared to infants without supplementation.

## Figures and Tables

**Table 1:** Standard and Experimental Infant Formula Nutrition Facts per 100 mL.

	Standard Formula	Experimental Formula
	100 mL (13.5%)	100 mL (13.5%)
Energy (kcal/kJ)	69/288	68/285
Proteins * (g)	1.35	1.35
Casein/whey (%)	40/60	40/60
Carbohydrates (g)	7.97	7.56
Lactose (g)	7.17	6.82
Maltodextrin (g)	0.8	0.7
Fat ^#^ (g)	3.5	3.5
Linoleic acid (LA, mg)	579	569
α-Linolenic acid (ALA, mg)	49	49
Arachidonic acid (AA, mg)	-	15.8
Docosahexaenoic acid (DHA, mg)	-	11.2

Powder diluted in water (13.5%) * With alpha-lactoalbumin (15% of total protein) and with Immunoglobulins. ^#^ With 22% of palmitic acid in beta position.

**Table 2:** Characteristics of the population.

Characteristics	SF		EF		BF		*p*
	*N*	Mean ± SD	*N*	Mean ± SD	*N*	Mean ± SD	
**Maternal characteristics**							
Age (years)	61	30.31 ^ab^ ± 6.53	70	29.97 ± 6.22 ^a^	45	33.24 ± 5.39 ^b^	**0.014**
Gestational age (months)	61	39.52 ± 1.29	70	39.26 ± 1.44	45	39.4 ± 1.3	0.55
Pre-pregnancy weight (kg)	56	65.43 ± 12.92	63	64.26 ± 12.52	43	65.66 ± 10.4	0.81
Pre-pregnancy BMI (kg/m^2^) (%)							0.76
Underweight	3	5.45	8	12.7	5	11.63	
Normal weight	29	52.73	29	46.03	23	53.49	
Overweight	14	25.45	15	23.81	11	25.58	
Obesity	9	16.36	11	17.46	4	9.3	
Education (%)							**<0.001**
Primary	14	22.58	15	21.43	1	2.22	
Secondary	18	29.03	25	35.71	4	8.89	
Professional	12	19.35	16	22.86	13	28.89	
Bachelor degree	18	29.03	14	20	27	60	
Smoking during pregnancy (Yes, %)	10	20.83	10	15.87	2	5.13	0.11
Edinburgh Scale (%)							0.42
No depression	49	79.03	53	76.81	39	86.67	
Probable depression	13	20.97	16	23.19	6	13.33	
**Infant characteristics**							
Sex, male (%)	37	59.68 ^ab^	44	62.86 ^a^	18	40.00 ^b^	**0.042**
Birth weight (kg)	61	3.34 ± 0.41	70	3.32 ± 0.5	45	3.35 ± 0.42	0.91
Birth length (cm)	61	50.67 ± 2.01	68	50.72 ± 2.1	45	50.62 ± 2.39	0.97
WAZ	61	0.04 ± 0.86	68	0.02 ± 0.97	44	0.13 ± 0.86	0.81
LAZ	61	0.58 ± 1.03	68	0.59 ± 1.08	44	0.71 ± 0.98	0.79
BMIZ	61	−0.39 ± 0.93	68	−0.39 ± 1.04	44	−0.37 ± 0.95	0.99

The presented values are means and proportions. Different superscript letters indicate differences among study groups according to ANOVA and Bonferroni post-hoc test. A chi-square test was applied to qualitative variables. Significance level was established at *p* < 0.05. SF, Standard Formula; EF, Experimental Formula; BF, Breastfeeding; WAZ, weight for age z-score; LAZ, length for age z-score; HAZ, height for age z-score.

**Table 3:** Associations between FADS genes and fatty acids levels in infants.

Fatty Acids and Gene	SNP	M/m	Standard Formula (*n* = 46) (*n* = 46)				Experimental Formula (*n* = 56) (*n* = 56)			Breastfeeding (*n* = 33) (*n* = 33)				
			β	*P*	βc	Pc	β	*P*	βc	Pc	β	*P*	βc	Pc
**C18:2*n*6 (LA)**														
*FADS1*	rs174537	G/T	−0.035	0.818	−0.132	0.408	−0.361	**0.006**	−0.376	0.005 *	−0.027	0.880	−0.094	0.687
*FADS1*	rs174545	C/G	−0.035	0.818	−0.132	0.408	−0.322	**0.015**	−0.351	**0.008**	0.026	0.888	−0.092	0.688
*FADS1*	rs174546	C/T	−0.035	0.818	−0.132	0.408	−0.322	**0.015**	−0.351	**0.008**	−0.027	0.880	−0.094	0.687
*FADS1*	rs174553	A/G	−0.035	0.818	−0.132	0.408	−0.322	**0.015**	−0.351	**0.008**	−0.027	0.880	−0.094	0.687
*FADS2*	rs1535	A/G	−0.035	0.818	−0.132	0.408	−0.346	**0.008**	−0.357	**0.008**	−0.027	0.880	−0.094	0.687
*FADS2*	rs174570	C/T	0.155	0.304	0.137	0.406	−0.272	**0.043**	−0.207	0.134	−0.106	0.558	−0.162	0.464
**C20:3*n*6 (DGLA)**														
*FADS2*	rs174570	C/T	−0.276	0.063	−0.230	0.167	−0.327	**0.014**	−0.288	**0.034**	−0.123	0.495	−0.046	0.815
*FADS2*	rs2072114	A/G	−0.056	0.709	−0.079	0.632	−0.368	0.005 *	−0.342	**0.014**	−0.090	0.618	−0.145	0.446
**C20:4*n*6 (AA)**														
*FADS1*	rs174537	G/T	−0.297	**0.045**	−0.224	0.155	−0.396	0.002 *	−0.440	0.001 *	0.023	0.897	0.035	0.876
*FADS1*	rs174545	C/G	−0.297	**0.045**	−0.224	0.155	−0.351	**0.007**	−0.375	**0.006**	0.046	0.803	0.036	0.873
*FADS1*	rs174546	C/T	−0.297	**0.045**	−0.224	0.155	−0.351	**0.007**	−0.375	**0.006**	0.023	0.897	0.035	0.876
*FADS1*	rs174548	C/G	−0.118	0.436	−0.073	0.653	−0.360	**0.006**	−0.367	**0.007**	0.052	0.773	0.034	0.878
*FADS1*	rs174553	A/G	−0.297	**0.045**	−0.224	0.155	−0.351	**0.007**	−0.375	**0.006**	0.023	0.897	0.035	0.876
*FADS2*	rs1535	A/G	−0.297	**0.045**	−0.224	0.155	−0.340	**0.010**	−0.374	**0.007**	0.023	0.897	0.035	0.876
*FADS2*	rs174570	C/T	−0.412	0.004 *	−0.347	**0.030**	−0.262	0.051	−0.237	**0.096**	−0.257	0.148	−0.187	0.379
*FADS2*	rs2072114	A/G	−0.077	0.613	−0.049	0.761	−0.502	<0.001 *	−0.522	<0.001 *	−0.059	0.746	−0.054	0.797
**C22:4*n*6 (AdA)**														
*FADS1*	rs174537	G/T	−0.350	**0.017**	−0.408	**0.010**	−0.346	**0.009**	−0.365	**0.006**	0.015	0.933	−0.042	0.855
*FADS1*	rs174545	C/G	−0.350	**0.017**	−0.408	**0.010**	−0.333	**0.011**	−0.330	**0.014**	0.005	0.979	−0.042	0.856
*FADS1*	rs174546	C/T	−0.350	**0.017**	−0.408	**0.010**	−0.333	**0.011**	−0.330	**0.014**	0.015	0.933	−0.042	0.855
*FADS1*	rs174548	C/G	−0.280	0.059	−0.372	**0.022**	−0.332	**0.012**	−0.317	**0.016**	−0.027	0.883	−0.140	0.542
*FADS1*	rs174553	A/G	−0.350	**0.017**	−0.408	**0.010**	−0.333	**0.011**	−0.330	**0.014**	0.015	0.933	−0.042	0.855
*FADS2*	rs1535	A/G	−0.350	**0.017**	−0.408	**0.010**	−0.348	**0.008**	−0.354	**0.009**	0.015	0.933	−0.042	0.855
*FADS2*	rs174570	C/T	−0.222	0.138	−0.244	0.147	−0.374	0.004 *	−0.337	**0.013**	−0.046	0.799	0.029	0.897
*FADS2*	rs2072114	A/G	0.060	0.693	0.025	0.883	−0.362	**0.006**	−0.302	**0.032**	0.011	0.953	−0.019	0.931
**C22:5*n*6 (DPAn6)**														
*FADS1*	rs174545	C/G	−0.111	0.463	−0.054	0.739	−0.255	0.056	−0.288	**0.038**	0.102	0.577	0.068	0.742
*FADS1*	rs174546	C/T	−0.111	0.463	−0.054	0.739	−0.255	0.056	−0.288	**0.038**	0.100	0.578	0.068	0.741
*FADS1*	rs174553	A/G	−0.111	0.463	−0.054	0.739	−0.255	0.056	−0.288	**0.038**	0.100	0.578	0.068	0.741
**C18:3*n*3 (ALA)**														
*FADS2*	rs174570	C/T	0.303	**0.040**	0.279	0.079	−0.040	0.772	−0.042	0.765	−0.061	0.736	−0.089	0.665
**C20:5*n*3 (EPA)**														
*FADS1*	rs174537	G/T	−0.287	0.053	−0.315	0.057	−0.249	0.065	−0.331	**0.017**	0.113	0.530	0.269	0.243
*FADS1*	rs174545	C/G	−0.287	0.053	−0.315	0.057	−0.225	0.093	0.310	**0.025**	0.162	0.374	0.274	0.236
*FADS1*	rs174546	C/T	−0.287	0.053	−0.315	0.057	−0.225	0.093	0.310	**0.025**	0.113	0.530	0.269	0.243
*FADS1*	rs174548	C/G	−0.238	0.112	−0.284	0.093	−0.247	0.064	−0.303	**0.026**	0.158	0.380	0.296	0.198
*FADS1*	rs174553	A/G	−0.287	0.053	−0.315	0.057	−0.225	0.093	−0.310	**0.025**	0.113	0.530	0.269	0.243
**GLA:LA (D6D)**														
*FADS2*	rs174570	C/T	−0.394	**0.007**	−0.338	**0.037**	−0.049	0.722	−0.127	0.361	−0.078	0.670	0.007	0.973
**DGLA:LA (D6D)**														
*FADS1*	rs174537	G/T	−0.24	**0.010**	−0.17	0.28	0.06	0.62	−0.01	0.94	0.09	0.62	0.19	0.38
*FADS1*	rs174545	C/G	−0.24	**0.010**	−0.17	0.28	0.04	0.72	−0.03	0.81	0.07	0.71	0.19	0.40
*FADS1*	rs174546	C/T	−0.24	**0.010**	−0.17	0.28	0.04	0.72	−0.03	0.81	0.09	0.62	0.19	0.38
*FADS1*	rs174553	A/G	−0.24	**0.010**	−0.17	0.28	0.04	0.72	−0.03	0.81	0.09	0.62	0.19	0.38
*FADS2*	rs174570	C/T	−0.40	**0.006**	−0.34	**0.032**	−0.21	0.12	−0.20	0.15	−0.08	0.64	0.01	0.93
*FADS2*	rs2072114	A/G	−0.18	0.22	−0.14	0.36	−0.29	**0.026**	−0.26	0.06	−0.09	0.59	−0.14	0.47
**AA:LA (D6D + D5D)**														
*FADS1*	rs174537	G/T	−0.36	**0.013**	−0.28	0.06	−0.22	0.09	−0.26	0.06	0.04	0.81	0.09	0.66
*FADS1*	rs174545	C/G	−0.36	**0.013**	−0.28	0.06	−0.20	0.13	−0.21	0.13	0.04	0.83	0.09	0.67
*FADS1*	rs174546	C/T	−0.36	**0.013**	−0.28	0.06	−0.20	0.13	−0.21	0.13	0.04	0.81	0.09	0.66
*FADS1*	rs174548	C/G	−0.18	0.24	−0.15	0.36	−0.27	**0.045**	−0.26	0.06	−0.01	0.95	−0.05	0.83
*FADS1*	rs174553	A/G	−0.36	**0.013**	−0.28	0.06	−0.20	0.13	−0.21	0.13	0.04	0.81	0.09	0.66
*FADS2*	rs1535	A/G	−0.36	**0.013**	−0.28	0.06	−0.17	0.18	−0.20	0.14	0.04	0.81	0.09	0.66
*FADS2*	rs174570	C/T	−0.47	0.001 *	−0.41	**0.007**	−0.13	0.32	−0.14	0.32	−0.24	0.16	−0.12	0.54
*FADS2*	rs2072114	A/G	−0.16	0.27	−0.12	0.43	−0.43	0.001 *	−0.450	0.001 *	−0.07	0.67	−0.05	0.79
**AA:DGLA (D5D)**														
*FADS1*	rs174548	C/G	−0.226	0.132	−0.160	0.333	−0.269	**0.043**	−0.285	**0.041**	−0.133	0.462	−0.108	0.636
*FADS2*	rs1535	A/G	−0.195	0.195	−0.141	0.386	−0.261	0.050	−0.283	**0.049**	−0.072	0.691	−0.071	0.757
**C22:6*n*3 (DHA)**														
*FADS1*	rs174537	G/T	−0.257	0.085	−0.303	0.054	−0.393	0.003 *	−0.415	0.002 *	0.057	0.753	0.176	0.432
*FADS1*	rs174545	C/G	−0.257	0.085	−0.303	0.054	−0.341	**0.010**	−0.339	**0.013**	0.089	0.629	0.177	0.429
*FADS1*	rs174546	C/T	−0.257	0.085	−0.303	0.054	−0.341	**0.010**	−0.339	**0.013**	0.057	0.753	0.176	0.432
*FADS1*	rs174548	C/G	−0.144	0.339	−0.211	0.192	−0.338	**0.010**	−0.328	**0.015**	0.101	0.577	0.164	0.463
*FADS1*	rs174553	A/G	−0.257	0.085	−0.303	0.054	−0.341	**0.010**	−0.339	**0.013**	0.057	0.753	0.176	0.432
*FADS2*	rs1535	A/G	−0.257	0.085	−0.303	0.054	−0.342	**0.009**	−0.354	**0.010**	0.057	0.753	0.176	0.432
*FADS2*	rs174570	C/T	−0.233	0.120	−0.258	0.114	−0.265	**0.048**	−0.238	0.092	−0.352	**0.045**	−0.274	0.196
*FADS2*	rs2072114	A/G	0.071	0.637	0.056	0.732	−0.289	**0.029**	−0.244	0.093	−0.081	0.654	−0.017	0.934
**EPA:ALA (D6D + D5D)**														
*FADS1*	rs174537	G/T	−0.37	**0.010**	−0.35	**0.022**	−0.13	0.32	−0.19	0.15	0.08	0.65	−0.04	0.83
*FADS1*	rs174545	C/G	−0.37	**0.010**	−0.35	**0.022**	−0.08	0.52	−0.14	0.30	0.06	0.71	−0.04	0.84
*FADS1*	rs174546	C/T	−0.37	**0.010**	−0.35	**0.022**	−0.08	0.52	−0.14	0.30	0.08	0.65	−0.04	0.83
*FADS1*	rs174553	A/G	−0.37	**0.010**	−0.35	**0.022**	−0.08	0.52	−0.14	0.30	0.08	0.65	−0.04	0.83
*FADS2*	rs1535	A/G	−0.37	**0.010**	−0.35	**0.022**	−0.07	0.61	−0.13	0.34	0.08	0.65	−0.04	0.83
*FADS2*	rs174570	C/T	−0.37	**0.010**	−0.37	**0.019**	−0.06	0.61	−0.07	0.58	−0.14	0.43	−0.09	0.65

Associations between SNPs and FAs were determined using linear regression analysis. βc and Pc are values corrected for potential confounders such as maternal age, maternal education, smoking and infant gender. SNPs were coded according to minor allele count and analyzed as a numeric variable. “β” = beta per minor allele standardized per the major allele. *p*-values < 0.05 are highlighted in bold and significant associations that persisted after Bonferroni corrections are additionally denoted by asterisks (* *p* < 0.005). M: Major allele; m: minor allele; SNP, single nucleotide polymorphism; LA: Linoleic Acid; GLA: gamma-linolenic acid; DGLA: dihomo-gamma-linolenic acid; AA: Arachidonic Acid; AdA: adrenic acid; DPA*n*6: docosapentaenoic acid *n*6; ALA: alpha-linolenic Acid; EPA: eicosapentaenoic acid; DHA: docosahexaenoic Acid.

**Table 4:** Fatty acids according to infant SNPs and study group.

Fatty Acids and Gene	SNP	*M/m*	MM							Mm + mm						
			Standard Formula		Experimental Formula		Breastfeeding		*p*	Standard Formula		Experimental Formula		Breastfeeding		*p*
			*N*	Mean ± SD	*N*	Mean ± SD	*N*	Mean ± SD		*N*	Mean ± SD	*N*	Mean ± SD	*N*	Mean ± SD	
**C20:4*n*6 (AA)**																
*FADS1*	rs174537	G/T	30	2.0000 ± 0.64 ^a^	41	2.4900 ± 0.53 ^b^	18	2.9200 ± 0.79 ^b^	<0.001 *	31	1.7300 ± 0.53 ^a^	25	2.0000 ± 0.62 ^a^	20	2.7300 ± 0.73 ^b^	<0.001 *
*FADS1*	rs174545	C/G	30	2.0000 ± 0.64 ^a^	40	2.4900 ± 0.54 ^b^	17	2.9000 ± 0.81 ^b^	<0.001 *	31	1.7300 ± 0.53 ^a^	27	2.0500 ± 0.63 ^a^	20	2.7300 ± 0.73 ^b^	<0.001 *
*FADS1*	rs174546	C/T	30	2.0000 ± 0.64 ^a^	40	2.4900 ± 0.54 ^b^	18	2.9200 ± 0.79 ^b^	<0.001 *	31	1.7300 ± 0.53 ^a^	27	2.0500 ± 0.63 ^a^	20	2.7300 ± 0.73 ^b^	<0.001 *
*FADS1*	rs174548	C/G	33	1.9200 ± 0.63 ^a^	40	2.5000 ± 0.54 ^b^	21	2.8700 ± 0.78 ^b^	<0.001 *	28	1.8000 ± 0.56 ^a^	27	2.0500 ± 0.62 ^a^	17	2.7500 ± 0.73 ^b^	<0.001 *
*FADS1*	rs174553	A/G	30	2.0000 ± 0.64 ^a^	40	2.4900 ± 0.54 ^b^	18	2.9200 ± 0.79^b^	<0.001 *	31	1.7300 ± 0.53 ^a^	27	2.0500 ± 0.63 ^a^	20	2.7300 ± 0.73 ^b^	<0.001 *
*FADS2*	rs1535	A/G	30	2.0000 ± 0.64 ^a^	40	2.4900 ± 0.54 ^b^	18	2.9200 ± 0.79 ^b^	<0.001 *	31	1.7300 ± 0.53 ^a^	27	2.0600 ± 0.63 ^a^	20	2.7300 ± 0.73 ^b^	<0.001 *
*FADS2*	rs174570	C/T	44	1.9600 ± 0.60 ^a^	53	2.4100 ± 0.58 ^b^	28	2.9500 ± 0.75 ^c^	<0.001 *	17	1.6200 ± 0.52 ^a^	13	1.8900 ± 0.57 ^ab^	10	2.4300 ± 0.64 ^b^	**0.006**
*FADS2*	rs2072114	A/G	47	1.8700 ± 0.61 ^a^	54	2.4400 ± 0.53 ^b^	30	2.8500 ± 0.76 ^b^	<0.001 *	14	1.8400 ± 0.56 ^a^	13	1.8100 ± 0.69 ^a^	8	2.6800 ± 0.75^b^	**0.007**
**C22:6*n*3 (DHA)**																
*FADS1*	rs174537	G/T	30	0.5100 ± 0.24 ^a^	41	0.9900 ± 0.22 ^b^	18	1.2300 ± 0.42 ^b^	<0.001 *	31	0.4500 ± 0.25 ^a^	25	0.7900 ± 0.27 ^b^	20	1.1800 ± 0.46 ^c^	<0.001 *
*FADS1*	rs174545	C/G	30	0.5100 ± 0.24 ^a^	40	0.9900 ± 0.22 ^b^	17	1.2000 ± 0.43 ^b^	<0.001 *	31	0.4500 ± 0.25 ^a^	27	0.8200 ± 0.28 ^b^	20	1.1800 ± 0.46 ^c^	<0.001 *
*FADS1*	rs174546	C/T	30	0.5100 ± 0.24 ^a^	40	0.9900 ± 0.22 ^b^	18	1.2300 ± 0.42 ^b^	<0.001 *	31	0.4500 ± 0.25 ^a^	27	0.8200 ± 0.28 ^b^	20	1.1800 ± 0.46 ^c^	<0.001 *
*FADS1*	rs174548	C/G	33	0.4900 ± 0.24 ^a^	40	0.9900 ± 0.22 ^b^	21	1.2000 ± 0.43^b^	<0.001 *	28	0.4600 ± 0.26 ^a^	27	0.8200 ± 0.29 ^b^	17	1.2000 ± 0.45 ^c^	<0.001 *
*FADS1*	rs174553	A/G	30	0.5100 ± 0.24 ^a^	40	0.9900 ± 0.22 ^b^	18	1.2300 ± 0.42 ^b^	<0.001 *	31	0.4500 ± 0.25 ^a^	27	0.8200 ± 0.28 ^b^	20	1.1800 ± 0.46 ^c^	<0.001 *
*FADS2*	rs1535	A/G	30	0.5100 ± 0.24 ^a^	40	0.9900 ± 0.22 ^b^	18	1.2300 ± 0.42 ^b^	<0.001 *	31	0.4500 ± 0.25 ^a^	27	0.8200 ± 0.28 ^b^	20	1.1800 ± 0.46 ^c^	<0.001 *
*FADS2*	rs174570	C/T	44	0.5000 ± 0.25 ^a^	53	0.9500 ± 0.22 ^b^	28	1.3000 ± 0.42 ^c^	<0.001 *	17	0.4200 ± 0.21 ^a^	13	0.7700 ± 0.33 ^b^	10	0.9200 ± 0.35 ^b^	<0.001 *
*FADS2*	rs2072114	A/G	47	0.4700 ± 0.24 ^a^	54	0.9600 ± 0.25 ^b^	30	1.2300 ± 0.45 ^b^	<0.001 *	14	0.5100 ± 0.28 ^a^	13	0.7600 ± 0.26 ^b^	8	1.0900 ± 0.38 ^c^	<0.001 *

Values are means ± standard deviations. The general linear model and Bonferroni post-hoc test were applied. The analysis was corrected for potential confounders such as pre-gestational IMC, smoking, education and age of mother and infant gender. *p*-values < 0.05 are highlighted in bold and significant differences that persisted after Bonferroni corrections are additionally denoted by asterisks (* *p* < 0.005). Different superscript letter indicate which groups are different from the others. M: Major allele; m: minor allele; SNP, single nucleotide polymorphism; AA: Arachidonic Acid; DHA: docosahexaenoic Acid.

## Supplementary Materials

The following are available online at https://www.mdpi.com/2072-6643/11/3/602/s1, Table S1: The genetic variants studied within the FADS genes, Table S2: Fatty acid content in infant formulas, Table S3: Associations between FADS genes and fatty acid levels in infants, Table S4: Fatty acids and enzymatic indexes according to infant SNPs and study group.
