# Supplementary material for: The Effect of an Infant Formula Supplemented with AA and DHA on Fatty Acid Levels of Infants with Different FADS Genotypes: The COGNIS Study
Source: Nutrients. 2019 Mar 12;11(3):602. doi: 10.3390/nu11030602 (PMC6470942; doi:10.3390/nu11030602)
Supplement: Supplementary file 1 [file nutrients-11-00602-s001.pdf]

Table S1. The genetic variants studied within the FADS genes.

Gene	SNP	Alleles	Standard Formula				Experimental Formula				Breastfeeding			
			N	MAF (%)	HWE	Missingness (%)	N	MAF (%)	HWE	Missingness (%)	N	MAF (%)	HWE	Missingness (%)
FADS1	rs174537	G/T	62	50.0	0.480	0.0	69	39.1	0.170	1.4	45	51.1	0.080	0.0
FADS1	rs174545	C/G	62	50.0	0.480	0.0	70	41.4	0.300	0.0	44	52.3	0.090	2.2
FADS1	rs174546	C/T	62	50.0	0.480	0.0	70	41.4	0.300	0.0	45	51.1	0.030	0.0
FADS1	rs174548	C/G	62	45.2	0.200	0.0	70	41.4	0.300	0.0	45	44.4	0.540	0.0
FADS1	rs174553	A/G	62	50.0	0.480	0.0	70	41.4	0.300	0.0	45	51.1	0.080	0.0
FADS2	rs1535	A/G	62	50.0	0.480	0.0	70	41.4	0.300	0.0	45	51.1	0.080	0.0
FADS2	rs174570	C/T	62	27.4	0.190	0.0	69	20.3	0.820	1.4	45	26.7	0.060	0.0
FADS2	rs2072114	A/G	62	22.6	0.320	0.0	70	18.6	0.390	0.0	45	22.2	0.120	0.0

[1] Values are proportions unless otherwise stated. HWE: Hardy-Weinberg Equilibrium was analyzed by chi-square tests and denotes P-values of deviation. MAF, Minor allele frequency.

Table S2. Fatty acid content in infant formulas

	Standard Formula 100 ml (DIL. 13.5%)			Experimental Formula 100 ml (DIL. 13.5%)				
	OMEGA FATS	BETAPOL		OMEGA FATS	BETAPOL	AA oil market	DHA oil lipid	
	90%	10%	%	88.08%	10%	1.12%	0.80%	%
**C8:0**	0.5	1.1	0.6	0.5	1.1	0.0	0.0	0.6
**C10:0**	0.5	1.0	0.6	0.5	1.0	0.0	0.0	0.5
**C12:0**	7.4	12.0	7.9	7.4	12.0	0.0	0.0	7.7
**C14:0**	3.1	4.0	3.2	3.1	4.0	0.0	0.0	3.1
**C16:0**	22.8	22.5	22.8	22.8	22.5	0.0	0.0	22.3
**C16:0 β**	4.1	10.1	4.7	4.1	10.1	0.0	0.0	4.6
**C18:0**	3.4	4.0	3.5	3.4	4.0	0.0	0.0	3.4
**C18:1 cis**	41.0	40.0	40.9	41.0	40.0	0.0	0.0	40.1
**C18:2**	17.0	12.0	16.5	17.0	12.0	0.0	0.0	16.2
**C18:3**	1.4	1.3	1.4	1.4	1.3	0.0	0.0	1.4
**AA**	0.0	0.0		0.0	0.0	40.0	0.0	0.45
**DHA**	0.0	0.0		0.0	0.0	0.0	40.0	0.32
**Others**			2.8					3.9
**C18:2/C18:3**	12.1	9.2	11.9	12.1	9.2	0.0	0.0	11.9
**C12:0+C14:0**	10.5	16.0	11.1	10.5	16.0	0.0	0.0	10.8
**SFA**	37.7	44.6	38.4	37.7	44.6	0.0	0.0	37.7
**MFA**	41.0	40.0	40.9	41.0	40.0	0.0	0.0	40.1
**PUFA**	18.4	13.3	17.9	18.4	13.3	40.0	40.0	18.3

OMEGA FATS content: 49% palm, 15% palm-kernel, 14% rapeseed, 13% sunflower, 9% oleic sunflower. BETAPOL content: palm, palm-kernel, sunflower and rapeseed. AA, Arachidonic acid; DHA, Docosahexaenoic acid; SFA, Saturated fatty acids; MFA, Monounsaturated fatty acids; PUFA, Polyunsaturated fatty acids.

Table S3. Associations between FADS genes and fatty acid levels in infants.

Fatty acids and Gene	SNP	*M/m*	Standard Formula (n=46)				Experimental Formula (n=56)				Breastfeeding (n=33)			
			β	P	βc	Pc	β	P	βc	Pc	β	P	βc	Pc
**C18:2n6 (LA)**														
FADS1	rs174537	G/T	-0.035	0.818	-0.132	0.408	-0.361	**0.006**	-0.376	**0.005***	-0.027	0.880	-0.094	0.687
FADS1	rs174545	C/G	-0.035	0.818	-0.132	0.408	-0.322	**0.015**	-0.351	**0.008**	0.026	0.888	-0.092	0.688
FADS1	rs174546	C/T	-0.035	0.818	-0.132	0.408	-0.322	**0.015**	-0.351	**0.008**	-0.027	0.880	-0.094	0.687
FADS1	rs174548	C/G	-0.058	0.701	-0.159	0.327	-0.218	0.103	-0.241	0.072	0.123	0.495	0.151	0.514
FADS1	rs174553	A/G	-0.035	0.818	-0.132	0.408	-0.322	**0.015**	-0.351	**0.008**	-0.027	0.880	-0.094	0.687
FADS2	rs1535	A/G	-0.035	0.818	-0.132	0.408	-0.346	**0.008**	-0.357	**0.008**	-0.027	0.880	-0.094	0.687
FADS2	rs174570	C/T	0.155	0.304	0.137	0.406	-0.272	**0.043**	-0.207	0.134	-0.106	0.558	-0.162	0.464
FADS2	rs2072114	A/G	0.177	0.240	-0.225	0.178	-0.182	0.176	-0.181	0.206	0.012	0.947	-0.017	0.938
**C18:3n6 (GLA)**														
FADS1	rs174537	G/T	-0.236	0.114	-0.236	0.148	-0.121	0.374	-0.207	0.137	0.094	0.608	0.191	0.399
FADS1	rs174545	C/G	-0.236	0.114	-0.236	0.148	-0.119	0.377	-0.216	0.120	0.086	0.644	0.187	0.413
FADS1	rs174546	C/T	-0.236	0.114	-0.236	0.148	-0.119	0.377	-0.216	0.120	0.094	0.608	0.191	0.399
FADS1	rs174548	C/G	-0.141	0.352	-0.176	0.292	-0.171	0.204	-0.242	0.076	0.118	0.518	0.146	0.521
FADS1	rs174553	A/G	-0.236	0.114	-0.236	0.148	-0.119	0.377	-0.216	0.120	0.094	0.608	0.191	0.399
FADS2	rs1535	A/G	-0.236	0.114	-0.236	0.148	-0.102	0.450	-0.207	0.141	0.094	0.608	0.191	0.399
FADS2	rs174570	C/T	-0.244	0.103	-0.207	0.221	-0.191	0.159	-0.235	0.093	-0.117	0.525	-0.039	0.859
FADS2	rs2072114	A/G	-0.076	0.616	-0.121	0.466	-0.070	0.603	-0.202	0.164	-0.123	0.501	-0.116	0.588
**C20:3n6 (DGLA)**														
FADS1	rs174537	G/T	-0.121	0.422	-0.095	0.558	-0.191	0.158	-0.227	0.096	0.181	0.312	0.171	0.399
FADS1	rs174545	C/G	-0.121	0.422	-0.095	0.558	-0.159	0.237	-0.170	0.212	0.160	0.382	0.168	0.409
FADS1	rs174546	C/T	-0.121	0.422	-0.095	0.558	-0.159	0.237	-0.170	0.212	0.181	0.312	0.171	0.399
FADS1	rs174548	C/G	0.064	0.671	0.055	0.739	-0.149	0.270	-0.144	0.284	0.238	0.182	0.234	0.244
FADS1	rs174553	A/G	-0.121	0.422	-0.095	0.558	-0.159	0.237	-0.170	0.212	0.181	0.312	0.171	0.399
FADS2	rs1535	A/G	-0.121	0.422	-0.095	0.558	-0.136	0.313	-0.152	0.270	0.181	0.312	0.171	0.399
FADS2	rs174570	C/T	-0.276	0.063	-0.230	0.167	-0.327	**0.014**	-0.288	**0.034**	-0.123	0.495	-0.046	0.815
FADS2	rs2072114	A/G	-0.056	0.709	-0.079	0.632	-0.368	**0.005***	-0.342	**0.014**	-0.090	0.618	-0.145	0.446
**C20:4n6 (AA)**														
FADS1	rs174537	G/T	-0.297	**0.045**	-0.224	0.155	-0.396	**0.002***	-0.440	**0.001***	0.023	0.897	0.035	0.876
FADS1	rs174545	C/G	-0.297	**0.045**	-0.224	0.155	-0.351	**0.007**	-0.375	**0.006**	0.046	0.803	0.036	0.873
FADS1	rs174546	C/T	-0.297	**0.045**	-0.224	0.155	-0.351	**0.007**	-0.375	**0.006**	0.023	0.897	0.035	0.876
FADS1	rs174548	C/G	-0.118	0.436	-0.073	0.653	-0.360	**0.006**	-0.367	**0.007**	0.052	0.773	0.034	0.878
FADS1	rs174553	A/G	-0.297	**0.045**	-0.224	0.155	-0.351	**0.007**	-0.375	**0.006**	0.023	0.897	0.035	0.876
FADS2	rs1535	A/G	-0.297	**0.045**	-0.224	0.155	-0.340	**0.010**	-0.374	**0.007**	0.023	0.897	0.035	0.876
FADS2	rs174570	C/T	-0.412	**0.004***	-0.347	**0.030**	-0.262	0.051	-0.237	**0.096**	-0.257	0.148	-0.187	0.379

FADS2	rs2072114	A/G	-0.077	0.613	-0.049	0.761	-0.502	**<0.001***	-0.522	**<0.001***	-0.059	0.746	-0.054	0.797
**C22:4n6 (AdA)**														
FADS1	rs174537	G/T	-0.350	**0.017**	-0.408	**0.010**	-0.346	**0.009**	-0.365	**0.006**	0.015	0.933	-0.042	0.855
FADS1	rs174545	C/G	-0.350	**0.017**	-0.408	**0.010**	-0.333	**0.011**	-0.330	**0.014**	0.005	0.979	-0.042	0.856
FADS1	rs174546	C/T	-0.350	**0.017**	-0.408	**0.010**	-0.333	**0.011**	-0.330	**0.014**	0.015	0.933	-0.042	0.855
FADS1	rs174548	C/G	-0.280	0.059	-0.372	**0.022**	-0.332	**0.012**	-0.317	**0.016**	-0.027	0.883	-0.140	0.542
FADS1	rs174553	A/G	-0.350	**0.017**	-0.408	**0.010**	-0.333	**0.011**	-0.330	**0.014**	0.015	0.933	-0.042	0.855
FADS2	rs1535	A/G	-0.350	**0.017**	-0.408	**0.010**	-0.348	**0.008**	-0.354	**0.009**	0.015	0.933	-0.042	0.855
FADS2	rs174570	C/T	-0.222	0.138	-0.244	0.147	-0.374	**0.004***	-0.337	**0.013**	-0.046	0.799	0.029	0.897
FADS2	rs2072114	A/G	0.060	0.693	0.025	0.883	-0.362	**0.006**	-0.302	**0.032**	0.011	0.953	-0.019	0.931
**C22:5n6 (DPAn6)**														
FADS1	rs174537	G/T	-0.111	0.463	-0.054	0.739	-0.177	0.192	-0.221	0.113	0.100	0.578	0.068	0.741
FADS1	rs174545	C/G	-0.111	0.463	-0.054	0.739	-0.255	0.056	-0.288	**0.038**	0.102	0.577	0.068	0.742
FADS1	rs174546	C/T	-0.111	0.463	-0.054	0.739	-0.255	0.056	-0.288	**0.038**	0.100	0.578	0.068	0.741
FADS1	rs174548	C/G	-0.018	0.906	0.036	0.829	-0.232	0.083	-0.238	0.085	0.104	0.563	0.044	0.831
FADS1	rs174553	A/G	-0.111	0.463	-0.054	0.739	-0.255	0.056	-0.288	**0.038**	0.100	0.578	0.068	0.741
FADS2	rs1535	A/G	-0.111	0.463	-0.054	0.739	-0.197	0.142	-0.239	0.092	0.100	0.578	0.068	0.741
FADS2	rs174570	C/T	-0.258	0.084	-0.215	0.195	-0.128	0.348	-0.208	0.138	-0.005	0.980	0.115	0.559
FADS2	rs2072114	A/G	0.008	0.956	0.056	0.732	-0.204	0.129	-0.285	0.051	-0.100	0.579	-0.145	0.447
**C18:3n3 (ALA)**														
FADS1	rs174537	G/T	0.224	0.135	0.160	0.305	0.024	0.858	0.060	0.667	-0.089	0.622	0.087	0.684
FADS1	rs174545	C/G	0.224	0.135	0.160	0.305	-0.027	0.844	-0.004	0.979	-0.040	0.829	0.091	0.677
FADS1	rs174546	C/T	0.224	0.135	0.160	0.305	-0.027	0.844	-0.004	0.979	-0.089	0.622	0.087	0.684
FADS1	rs174548	C/G	0.002	0.987	-0.022	0.889	0.018	0.892	0.037	0.789	-0.013	0.945	0.209	0.325
FADS1	rs174553	A/G	0.224	0.135	0.160	0.305	-0.027	0.844	-0.004	0.979	-0.089	0.622	0.087	0.684
FADS2	rs1535	A/G	0.224	0.135	0.160	0.305	-0.019	0.886	0.017	0.902	-0.089	0.622	0.087	0.684
FADS2	rs174570	C/T	0.303	**0.040**	0.279	0.079	-0.040	0.772	-0.042	0.765	-0.061	0.736	-0.089	0.665
FADS2	rs2072114	A/G	-0.049	0.747	-0.123	0.436	0.030	0.823	0.026	0.859	-0.186	0.300	-0.113	0.572
**C20:5n3 (EPA)**														
FADS1	rs174537	G/T	-0.287	0.053	-0.315	0.057	-0.249	0.065	-0.331	**0.017**	0.113	0.530	0.269	0.243
FADS1	rs174545	C/G	-0.287	0.053	-0.315	0.057	-0.225	0.093	0.310	**0.025**	0.162	0.374	0.274	0.236
FADS1	rs174546	C/T	-0.287	0.053	-0.315	0.057	-0.225	0.093	0.310	**0.025**	0.113	0.530	0.269	0.243
FADS1	rs174548	C/G	-0.238	0.112	-0.284	0.093	-0.247	0.064	-0.303	**0.026**	0.158	0.380	0.296	0.198
FADS1	rs174553	A/G	-0.287	0.053	-0.315	0.057	-0.225	0.093	-0.310	**0.025**	0.113	0.530	0.269	0.243
FADS2	rs1535	A/G	-0.287	0.053	-0.315	0.057	-0.176	0.191	-0.260	0.065	0.113	0.530	0.269	0.243
FADS2	rs174570	C/T	-0.129	0.392	-0.154	0.375	-0.207	0.126	-0.228	0.108	-0.313	0.076	-0.295	0.178
FADS2	rs2072114	A/G	0.138	0.359	0.161	0.345	0.084	0.534	0.044	0.767	-0.045	0.805	0.026	0.907
**C22:5n3 (DPAn3)**														

FADS1	rs174537	G/T	0.009	0.952	0.092	0.574	-0.022	0.860	-0.055	0.696	0.036	0.841	0.055	0.803
FADS1	rs174545	C/G	0.009	0.952	0.092	0.574	0.003	0.982	-0.026	0.849	0.044	0.812	0.055	0.802
FADS1	rs174546	C/T	0.009	0.952	0.092	0.574	0.003	0.982	-0.026	0.849	0.036	0.841	0.055	0.803
FADS1	rs174548	C/G	0.046	0.761	0.115	0.487	-0.037	0.784	-0.059	0.665	0.095	0.599	0.071	0.750
FADS1	rs174553	A/G	0.009	0.952	0.092	0.574	0.003	0.982	-0.026	0.849	0.036	0.841	0.055	0.803
FADS2	rs1535	A/G	0.009	0.952	0.092	0.574	0.076	0.575	0.064	0.651	0.036	0.841	0.055	0.803
FADS2	rs174570	C/T	-0.111	0.464	-0.046	0.787	-0.095	0.487	-0.026	0.852	-0.107	0.554	-0.076	0.721
FADS2	rs2072114	A/G	0.072	0.636	0.110	0.505	-0.004	0.979	0.027	0.853	0.062	0.731	0.091	0.661
**GLA:LA (D6D)**														
FADS1	rs174537	G/T	-0.104	0.492	-0.009	0.957	-0.030	0.824	-0.061	0.661	0.197	0.273	0.212	0.312
FADS1	rs174545	C/G	-0.104	0.492	-0.009	0.957	-0.017	0.901	-0.015	0.915	0.158	0.389	0.213	0.312
FADS1	rs174546	C/T	-0.104	0.492	-0.009	0.957	-0.017	0.901	-0.015	0.915	0.197	0.273	0.212	0.312
FADS1	rs174548	C/G	0.109	0.471	0.170	0.286	-0.053	0.694	-0.038	0.777	0.195	0.278	0.180	0.392
FADS1	rs174553	A/G	-0.104	0.492	-0.009	0.957	-0.017	0.901	-0.015	0.915	0.197	0.273	0.212	0.312
FADS2	rs1535	A/G	-0.246	0.099	0.175	0.275	0.079	0.560	-0.020	0.885	0.091	0.620	0.198	0.382
FADS2	rs174570	C/T	-0.394	**0.007**	-0.338	**0.037**	-0.049	0.722	-0.127	0.361	-0.078	0.670	0.007	0.973
FADS2	rs2072114	A/G	-0.217	0.148	-0.204	0.207	0.024	0.857	-0.108	0.453	-0.116	0.528	-0.101	0.637
**DGLA:LA (D6D)**														
FADS1	rs174537	G/T	-0.24	**0.010**	-0.17	0.28	0.06	0.62	-0.01	0.94	0.09	0.62	0.19	0.38
FADS1	rs174545	C/G	-0.24	**0.010**	-0.17	0.28	0.04	0.72	-0.03	0.81	0.07	0.71	0.19	0.40
FADS1	rs174546	C/T	-0.24	**0.010**	-0.17	0.28	0.04	0.72	-0.03	0.81	0.09	0.62	0.19	0.38
FADS1	rs174548	C/G	-0.12	0.43	-0.09	0.60	-0.06	0.67	-0.17	0.39	0.07	0.69	0.09	0.69
FADS1	rs174553	A/G	-0.24	**0.010**	-0.17	0.28	0.04	0.72	-0.03	0.81	0.09	0.62	0.19	0.38
FADS2	rs1535	A/G	-0.10	0.49	-0.01	0.96	0.02	0.89	0.01	0.96	0.20	0.27	0.21	0.31
FADS2	rs174570	C/T	-0.40	**0.006**	-0.34	**0.032**	-0.21	0.12	-0.20	0.15	-0.08	0.64	0.01	0.93
FADS2	rs2072114	A/G	-0.18	0.22	-0.14	0.36	-0.29	**0.026**	-0.26	0.06	-0.09	0.59	-0.14	0.47
**AA:LA (D6D+D5D)**														
FADS1	rs174537	G/T	-0.36	**0.013**	-0.28	0.06	-0.22	0.09	-0.26	0.06	0.04	0.81	0.09	0.66
FADS1	rs174545	C/G	-0.36	**0.013**	-0.28	0.06	-0.20	0.13	-0.21	0.13	0.04	0.83	0.09	0.67
FADS1	rs174546	C/T	-0.36	**0.013**	-0.28	0.06	-0.20	0.13	-0.21	0.13	0.04	0.81	0.09	0.66
FADS1	rs174548	C/G	-0.18	0.24	-0.15	0.36	-0.27	**0.045**	-0.26	0.06	-0.01	0.95	-0.05	0.83
FADS1	rs174553	A/G	-0.36	**0.013**	-0.28	0.06	-0.20	0.13	-0.21	0.13	0.04	0.81	0.09	0.66
FADS2	rs1535	A/G	-0.36	**0.013**	-0.28	0.06	-0.17	0.18	-0.20	0.14	0.04	0.81	0.09	0.66
FADS2	rs174570	C/T	-0.47	**0.001***	-0.41	**0.007**	-0.13	0.32	-0.14	0.32	-0.24	0.16	-0.12	0.54
FADS2	rs2072114	A/G	-0.16	0.27	-0.12	0.43	-0.43	**0.001***	-0.450	**0.001***	-0.07	0.67	-0.05	0.79
**AA:DGLA (D5D)**														
FADS1	rs174537	G/T	-0.195	0.195	-0.141	0.386	-0.252	0.061	-0.257	0.073	-0.072	0.691	-0.071	0.757
FADS1	rs174545	C/G	-0.195	0.195	-0.141	0.386	-0.240	0.072	-0.257	0.071	-0.018	0.920	-0.070	0.764

FADS1	rs174546	C/T	-0.195	0.195	-0.141	0.386	-0.240	0.072	-0.257	0.071	-0.072	0.691	-0.071	0.757
FADS1	rs174548	C/G	-0.226	0.132	-0.160	0.333	-0.269	**0.043**	-0.285	**0.041**	-0.133	0.462	-0.108	0.636
FADS1	rs174553	A/G	-0.195	0.195	-0.141	0.386	-0.240	0.072	-0.257	0.071	-0.072	0.691	-0.071	0.757
FADS2	rs1535	A/G	-0.195	0.195	-0.141	0.386	-0.261	0.050	-0.283	**0.049**	-0.072	0.691	-0.071	0.757
FADS2	rs174570	C/T	-0.127	0.401	-0.110	0.513	0.137	0.314	0.113	0.440	-0.022	0.904	-0.097	0.657
FADS2	rs2072114	A/G	-0.017	0.912	0.046	0.781	-0.129	0.339	-0.197	0.189	0.114	0.527	0.151	0.479
**C22:6n3 (DHA)**														
FADS1	rs174537	G/T	-0.257	0.085	-0.303	0.054	-0.393	**0.003***	-0.415	**0.002***	0.057	0.753	0.176	0.432
FADS1	rs174545	C/G	-0.257	0.085	-0.303	0.054	-0.341	**0.010**	-0.339	**0.013**	0.089	0.629	0.177	0.429
FADS1	rs174546	C/T	-0.257	0.085	-0.303	0.054	-0.341	**0.010**	-0.339	**0.013**	0.057	0.753	0.176	0.432
FADS1	rs174548	C/G	-0.144	0.339	-0.211	0.192	-0.338	**0.010**	-0.328	**0.015**	0.101	0.577	0.164	0.463
FADS1	rs174553	A/G	-0.257	0.085	-0.303	0.054	-0.341	**0.010**	-0.339	**0.013**	0.057	0.753	0.176	0.432
FADS2	rs1535	A/G	-0.257	0.085	-0.303	0.054	-0.342	**0.009**	-0.354	**0.010**	0.057	0.753	0.176	0.432
FADS2	rs174570	C/T	-0.233	0.120	-0.258	0.114	-0.265	**0.048**	-0.238	0.092	-0.352	**0.045**	-0.274	0.196
FADS2	rs2072114	A/G	0.071	0.637	0.056	0.732	-0.289	**0.029**	-0.244	0.093	-0.081	0.654	-0.017	0.934
**EPA:ALA (D6D+D5D)**														
FADS1	rs174537	G/T	-0.37	**0.010**	-0.35	**0.022**	-0.13	0.32	-0.19	0.15	0.08	0.65	-0.04	0.83
FADS1	rs174545	C/G	-0.37	**0.010**	-0.35	**0.022**	-0.08	0.52	-0.14	0.30	0.06	0.71	-0.04	0.84
FADS1	rs174546	C/T	-0.37	**0.010**	-0.35	**0.022**	-0.08	0.52	-0.14	0.30	0.08	0.65	-0.04	0.83
FADS1	rs174548	C/G	-0.18	0.23	-0.21	0.20	-0.13	0.34	-0.17	0.22	0.01	0.94	-0.19	0.37
FADS1	rs174553	A/G	-0.37	**0.010**	-0.35	**0.022**	-0.08	0.52	-0.14	0.30	0.08	0.65	-0.04	0.83
FADS2	rs1535	A/G	-0.37	**0.010**	-0.35	**0.022**	-0.07	0.61	-0.13	0.34	0.08	0.65	-0.04	0.83
FADS2	rs174570	C/T	-0.37	**0.010**	-0.37	**0.019**	-0.06	0.61	-0.07	0.58	-0.14	0.43	-0.09	0.65
FADS2	rs2072114	A/G	0.04	0.78	0.09	0.58	0.02	0.91	0.00	0.10	0.24	0.19	0.22	0.26

Associations between SNPs and FAs were determined using linear regression analysis. βc and Pc are values corrected for potential confounders such as maternal age, maternal education, smoking and infant gender. SNPs were coded according to minor allele count and analyzed as a numeric variable. "β" = beta per minor allele standardized per the major allele. P-values <0.05 are highlighted in bold and significant associations that persisted after Bonferroni corrections are additionally denoted by stars (*P<0.005). M: Major allele; m: minor allele; SNP, single nucleotide polymorphism; LA: Linoleic Acid; GLA: gamma-linolenic acid; DGLA: dihomo-gamma-linolenic acid; AA: Arachidonic Acid; AdA: adrenic acid; DPAn6: docosapentaenoic acid n6; ALA: alpha-linolenic Acid; EPA: eicosapentaenoic acid; DPAn3: docosapentaenoic acid n3; DHA: docosahexaenoic Acid.

Table S4. Fatty acids and enzymatic indexes according to infant SNPs and study group.

Fatty acids and Gene	SNP	M/m	MM							Mm+mm						
			SF		EF		BF		P	SF		EF		BF		P
			N	Mean ± SD	N	Mean ± SD	N	Mean ± SD		N	Mean ± SD	N	Mean ± SD	N	Mean ± SD	
**C18:2n6 (LA)**																
FADS1	rs174537	G/T	30	12.6600 ± 2.19	41	12.7000 ± 1.96	18	13.4300 ± 2.38	0.53	31	13.0300 ± 2.67[a]	25	11.2800 ± 1.76[b]	20	13.0400 ± 1.66[a]	**0.013**
FADS1	rs174545	C/G	30	12.6600 ± 2.19	40	12.6800 ± 1.98	17	13.2500 ± 2.31	0.69	31	13.0300 ± 2.67[ab]	27	11.4100 ± 1.76[a]	20	13.0400 ± 1.66[b]	**0.022**
FADS1	rs174546	C/T	30	12.6600 ± 2.19	40	12.6800 ± 1.98	18	13.4300 ± 2.38	0.52	31	13.0300 ± 2.67[ab]	27	11.4100 ± 1.76[a]	20	13.0400 ± 1.66[b]	**0.022**
FADS1	rs174548	C/G	33	12.7400 ± 2.22	40	12.5400 ± 2.03	21	13.1800 ± 2.51	0.68	28	12.9700 ± 2.69[ab]	27	11.6300 ± 1.80[a]	17	13.2800 ± 1.22[b]	**0.041**
FADS1	rs174553	A/G	30	12.6600 ± 2.19	40	12.6800 ± 1.98	18	13.4300 ± 2.38	0.52	31	13.0300 ± 2.67[ab]	27	11.4100 ± 1.76[a]	20	13.0400 ± 1.66[b]	**0.022**
FADS2	rs1535	A/G	30	12.6600 ± 2.19	40	12.7200 ± 1.99	18	13.4300 ± 2.38	0.53	31	13.0300 ± 2.67[a]	27	11.3600 ± 1.70[b]	20	13.0400 ± 1.66[a]	**0.017**
FADS2	rs174570	C/T	44	12.5700 ± 2.57	53	12.4800 ± 2.00	28	13.4300 ± 2.03	0.15	17	13.5700 ± 1.89[a]	13	10.8700 ± 1.43[b]	10	12.6500 ± 1.97[ab]	**0.007**
FADS2	rs2072114	A/G	47	12.5100 ± 2.50	54	12.3600 ± 1.95	30	13.3300 ± 2.24	0.17	14	13.9800 ± 1.81[a]	13	11.4000 ± 2.02[b]	8	12.8400 ± 0.73[ab]	**0.016**
**C18:3n6 (GLA)**																
FADS1	rs174537	G/T	30	0.1200 ± 0.07	41	0.1000 ± 0.04	18	0.1400 ± 0.09	0.11	31	0.1000 ± 0.03[a]	25	0.0900 ± 0.03[a]	20	0.1500 ± 0.09[b]	**<0.001***
FADS1	rs174545	C/G	30	0.1200 ± 0.07	40	0.1000 ± 0.04	17	0.1400 ± 0.09	0.12	31	0.1000 ± 0.03[a]	27	0.0900 ± 0.03[a]	20	0.1500 ± 0.09[b]	**<0.001***
FADS1	rs174546	C/T	30	0.1200 ± 0.07	40	0.1000 ± 0.04	18	0.1400 ± 0.09	0.12	31	0.1000 ± 0.03[a]	27	0.0900 ± 0.03[a]	20	0.1500 ± 0.09[b]	**<0.001***
FADS1	rs174548	C/G	33	0.1200 ± 0.07	40	0.1000 ± 0.04	21	0.1400 ± 0.08	0.16	28	0.1000 ± 0.03[a]	27	0.0900 ± 0.03[a]	17	0.1600 ± 0.10[b]	**<0.001***
FADS1	rs174553	A/G	30	0.1200 ± 0.07	40	0.1000 ± 0.04	18	0.1400 ± 0.09	0.12	31	0.1000 ± 0.03[a]	27	0.0900 ± 0.03[a]	20	0.1500 ± 0.09[b]	**<0.001***
FADS2	rs1535	A/G	30	0.1200 ± 0.07	40	0.1000 ± 0.04	18	0.1400 ± 0.09	0.11	31	0.1000 ± 0.03[a]	27	0.0900 ± 0.03[a]	20	0.1500 ± 0.09[b]	**<0.001***
FADS2	rs174570	C/T	44	0.1200 ± 0.07[ab]	53	0.1000 ± 0.04[a]	28	0.1600 ± 0.10[b]	**0.003***	17	0.0900 ± 0.02[a]	13	0.0900 ± 0.02[ab]	10	0.1200 ± 0.02[b]	**0.031**
FADS2	rs2072114	A/G	47	0.1100 ± 0.06[a]	54	0.1000 ± 0.03[a]	30	0.1500 ± 0.10[b]	**0.002***	14	0.1000 ± 0.03[a]	13	0.0900 ± 0.04[b]	8	0.1200 ± 0.03[ab]	0.16
**C20:3n6 (DGLA)**																
FADS1	rs174537	G/T	30	0.8100 ± 0.27	41	0.7100 ± 0.20	18	0.9300 ± 0.37	0.10	31	0.7900 ± 0.26[a]	25	0.6400 ± 0.28 [a]	20	1.0100 ± 0.31[b]	**<0.001***
FADS1	rs174545	C/G	30	0.8100 ± 0.27	40	0.7100 ± 0.20	17	0.9500 ± 0.37	0.08	31	0.7900 ± 0.26[a]	27	0.6500 ± 0.27[a]	20	1.0100 ± 0.31[b]	**<0.001***
FADS1	rs174546	C/T	30	0.8100 ± 0.27	40	0.7100 ± 0.20	18	0.9300 ± 0.37	0.10	31	0.7900 ± 0.26[a]	27	0.6500 ± 0.27[a]	20	1.0100 ± 0.31[b]	**<0.001***
FADS1	rs174548	C/G	33	0.7900 ± 0.28	40	0.7100 ± 0.20	21	0.9100 ± 0.35	0.10	28	0.8200 ± 0.25[a]	27	0.6500 ± 0.27[b]	17	1.0500 ± 0.31[a]	**<0.001***
FADS1	rs174553	A/G	30	0.8100 ± 0.27	40	0.7100 ± 0.20	18	0.9300 ± 0.37	0.10	31	0.7900 ± 0.26[a]	27	0.6500 ± 0.27[a]	20	1.0100 ± 0.31[b]	**<0.001***
FADS2	rs1535	A/G	30	0.8100 ± 0.27	40	0.7100 ± 0.20	18	0.9300 ± 0.37	0.09	31	0.7900 ± 0.26[a]	27	0.6600 ± 0.27[a]	20	1.0100 ± 0.31[b]	**<0.001***
FADS2	rs174570	C/T	44	0.8300 ± 0.28[ab]	53	0.7200 ± 0.22[a]	28	1.0100 ± 0.37[b]	**0.002***	17	0.7200 ± 0.23[a]	13	0.5200 ± 0.23[b]	10	0.8700 ± 0.21[a]	**0.001***
FADS2	rs2072114	A/G	47	0.8000 ± 0.28[a]	54	0.7200 ± 0.22[a]	30	1.0000 ± 0.35[b]	**0.002***	14	0.7900 ± 0.24[a]	13	0.5400 ± 0.23[b]	8	0.8900 ± 0.25[a]	**0.001***
**C20:4n6 (AA)**																
FADS1	rs174537	G/T	30	2.0000 ± 0.64[a]	41	2.4900 ± 0.53[b]	18	2.9200 ± 0.79[b]	**<0.001***	31	1.7300 ± 0.53[a]	25	2.0000 ± 0.62[a]	20	2.7300 ± 0.73[b]	**<0.001***
FADS1	rs174545	C/G	30	2.0000 ± 0.64[a]	40	2.4900 ± 0.54[b]	17	2.9000 ± 0.81[b]	**<0.001***	31	1.7300 ± 0.53[a]	27	2.0500 ± 0.63[a]	20	2.7300 ± 0.73[b]	**<0.001***

FADS1	rs174546	C/T	30	2.0000 ± 0.64^{a}	40	2.4900 ± 0.54^{b}	18	2.9200 ± 0.79^{b}	**<0.001***	31	1.7300 ± 0.53^{a}	27	2.0500 ± 0.63^{a}	20	2.7300 ± 0.73^{b}	**<0.001***
FADS1	rs174548	C/G	33	1.9200 ± 0.63^{a}	40	2.5000 ± 0.54^{b}	21	2.8700 ± 0.78^{b}	**<0.001***	28	1.8000 ± 0.56^{a}	27	2.0500 ± 0.62^{a}	17	2.7500 ± 0.73^{b}	**<0.001***
FADS1	rs174553	A/G	30	2.0000 ± 0.64^{a}	40	2.4900 ± 0.54^{b}	18	2.9200 ± 0.79^{b}	**<0.001***	31	1.7300 ± 0.53^{a}	27	2.0500 ± 0.63^{a}	20	2.7300 ± 0.73^{b}	**<0.001***
FADS2	rs1535	A/G	30	2.0000 ± 0.64^{a}	40	2.4900 ± 0.54^{b}	18	2.9200 ± 0.79^{b}	**<0.001***	31	1.7300 ± 0.53^{a}	27	2.0600 ± 0.63^{a}	20	2.7300 ± 0.73^{b}	**<0.001***
FADS2	rs174570	C/T	44	1.9600 ± 0.60^{a}	53	2.4100 ± 0.58^{b}	28	2.9500 ± 0.75^{c}	**<0.001***	17	1.6200 ± 0.52^{a}	13	1.8900 ± 0.57^{ab}	10	2.4300 ± 0.64^{b}	**0.006**
FADS2	rs2072114	A/G	47	1.8700 ± 0.61^{a}	54	2.4400 ± 0.53^{b}	30	2.8500 ± 0.76^{b}	**<0.001***	14	1.8400 ± 0.56^{a}	13	1.8100 ± 0.69^{a}	8	2.6800 ± 0.75^{b}	**0.007**
**C22:4n6 (AdA)**																
FADS1	rs174537	G/T	30	0.2200 ± 0.08^{a}	41	0.2400 ± 0.07^{a}	18	0.3000 ± 0.09^{b}	**0.004***	31	0.1800 ± 0.00^{a}	25	0.1800 ± 0.07^{a}	20	0.3000 ± 0.06^{b}	**<0.001***
FADS1	rs174545	C/G	30	0.2200 ± 0.08^{a}	40	0.2400 ± 0.07^{a}	17	0.3100 ± 0.09^{b}	**0.004***	31	0.1800 ± 0.05^{a}	27	0.1800 ± 0.07^{a}	20	0.3000 ± 0.06^{b}	**<0.001***
FADS1	rs174546	C/T	30	0.2200 ± 0.08^{a}	40	0.2400 ± 0.07^{a}	18	0.3000 ± 0.09^{b}	**0.004***	31	0.1800 ± 0.05^{a}	27	0.1800 ± 0.07^{a}	20	0.3000 ± 0.06^{b}	**<0.001***
FADS1	rs174548	C/G	33	0.2100 ± 0.08^{a}	40	0.2400 ± 0.07^{a}	21	0.3000 ± 0.09^{b}	**0.001***	28	0.1800 ± 0.06^{a}	27	0.1800 ± 0.07^{a}	17	0.2900 ± 0.06^{b}	**<0.001***
FADS1	rs174553	A/G	30	0.2200 ± 0.08^{a}	40	0.2400 ± 0.07^{a}	18	0.3000 ± 0.09^{b}	**0.004***	31	0.1800 ± 0.05^{a}	27	0.1800 ± 0.07^{a}	20	0.3000 ± 0.06^{b}	**<0.001***
FADS2	rs1535	A/G	30	0.2200 ± 0.08^{a}	40	0.2400 ± 0.07^{ab}	18	0.3000 ± 0.09^{b}	**0.004***	31	0.1800 ± 0.05^{a}	27	0.1800 ± 0.07^{a}	20	0.3000 ± 0.06^{b}	**<0.001***
FADS2	rs174570	C/T	44	0.2100 ± 0.08^{a}	53	0.2300 ± 0.07^{a}	28	0.3000 ± 0.08^{b}	**<0.001***	17	0.1800 ± 0.04^{a}	13	0.1500 ± 0.06^{a}	10	0.2900 ± 0.06^{b}	**<0.001***
FADS2	rs2072114	A/G	47	0.2000 ± 0.08^{a}	54	0.2300 ± 0.07^{a}	30	0.3000 ± 0.08^{b}	**<0.001***	14	0.2000 ± 0.05^{a}	13	0.1600 ± 0.06^{a}	8	0.3100 ± 0.07^{b}	**<0.001***
**C22:5n6 (DPAn6)**																
FADS1	rs174537	G/T	30	0.1100 ± 0.06^{a}	41	0.0800 ± 0.04^{b}	18	0.0700 ± 0.04^{b}	**0.007**	31	0.1000 ± 0.06^{a}	25	0.0700 ± 0.05^{b}	20	0.0700 ± 0.02^{ab}	**0.010**
FADS1	rs174545	C/G	30	0.1100 ± 0.06^{a}	40	0.0900 ± 0.05^{ab}	17	0.0700 ± 0.04^{b}	**0.011**	31	0.1000 ± 0.06^{a}	27	0.0700 ± 0.05^{b}	20	0.0700 ± 0.02^{ab}	**0.007**
FADS1	rs174546	C/T	30	0.1100 ± 0.06^{a}	40	0.0900 ± 0.05^{ab}	18	0.0700 ± 0.04^{b}	**0.010**	31	0.1000 ± 0.06^{a}	27	0.0700 ± 0.05^{b}	20	0.0700 ± 0.02^{ab}	**0.007**
FADS1	rs174548	C/G	33	0.1100 ± 0.06^{a}	40	0.0800 ± 0.05^{ab}	21	0.0700 ± 0.04^{b}	**0.012**	28	0.1100 ± 0.06^{a}	27	0.0700 ± 0.05^{b}	17	0.0700 ± 0.02^{ab}	**0.006**
FADS1	rs174553	A/G	30	0.1100 ± 0.06^{a}	40	0.0900 ± 0.05^{ab}	18	0.0700 ± 0.04^{b}	**0.010**	31	0.1000 ± 0.06^{a}	27	0.0700 ± 0.05^{b}	20	0.0700 ± 0.02^{ab}	**0.007**
FADS2	rs1535	A/G	30	0.1100 ± 0.06^{a}	40	0.0800 ± 0.05^{ab}	18	0.0700 ± 0.04^{b}	**0.009**	31	0.1000 ± 0.06^{a}	27	0.0700 ± 0.05^{b}	20	0.0700 ± 0.02^{ab}	**0.012**
FADS2	rs174570	C/T	44	0.1100 ± 0.06^{a}	53	0.0800 ± 0.05^{b}	28	0.0700 ± 0.03^{b}	**<0.001***	17	0.0900 ± 0.05	13	0.0700 ± 0.04	10	0.0700 ± 0.03	0.21
FADS2	rs2072114	A/G	47	0.1100 ± 0.06^{a}	54	0.0800 ± 0.05^{ab}	30	0.0700 ± 0.03^{b}	**0.008**	14	0.1100 ± 0.07^{a}	13	0.0600 ± 0.04^{b}	8	0.0600 ± 0.02^{ab}	**0.005***
**GLA:LA (D6D)**																
FADS1	rs174537	G/T	30	0.0100 ± 0.00	41	0.0000 ± 0.00	18	0.0100 ± 0.00	0.23	31	0.0000 ± 0.00^{a}	25	0.0000 ± 0.00^{ab}	20	0.0100 ± 0.00^{b}	**0.004***
FADS1	rs174545	C/G	30	0.0100 ± 0.01	40	0.0000 ± 0.00	17	0.0100 ± 0.01	0.23	31	0.0100 ± 0.00^{a}	27	0.0100 ± 0.00^{a}	20	0.0100 ± 0.01^{b}	**0.003***
FADS1	rs174546	C/T	30	0.0100 ± 0.01	40	0.0100 ± 0.00	18	0.0100 ± 0.01	0.26	31	0.0100 ± 0.00^{a}	27	0.0100 ± 0.00^{a}	20	0.0100 ± 0.01^{b}	**0.003***
FADS1	rs174548	C/G	33	0.0100 ± 0.01	40	0.0100 ± 0.00	21	0.0100 ± 0.01	0.37	28	0.0100 ± 0.00^{a}	27	0.0100 ± 0.00^{a}	17	0.0100 ± 0.01^{b}	**0.010**
FADS1	rs174553	A/G	30	0.0100 ± 0.01	40	0.0100 ± 0.00	18	0.0100 ± 0.01	0.26	31	0.0100 ± 0.00^{a}	27	0.0100 ± 0.00^{a}	20	0.0100 ± 0.01^{b}	**0.003***
FADS2	rs1535	A/G	30	0.0100 ± 0.01	40	0.0100 ± 0.00	18	0.0100 ± 0.01	0.23	31	0.0100 ± 0.00^{a}	27	0.0100 ± 0.00^{ab}	20	0.0100 ± 0.01^{b}	**0.004***
FADS2	rs174570	C/T	44	0.0100 ± 0.01^{ab}	53	0.0100 ± 0.00^{a}	28	0.0100 ± 0.01^{b}	**0.044**	17	0.0100 ± 0.00^{a}	13	0.0100 ± 0.00^{ab}	10	0.0100 ± 0.00^{b}	**0.022**
FADS2	rs2072114	A/G	47	0.0100 ± 0.01^{ab}	54	0.0100 ± 0.00^{a}	30	0.0100 ± 0.01^{b}	**0.036**	14	0.0100 ± 0.00	13	0.0100 ± 0.00	8	0.0100 ± 0.00	0.16
**DGLA:LA (D6D)**																

FADS1	rs174537	G/T	30	0.0600 ± 0.02	41	0.0500 ± 0.01	18	0.0700 ± 0.03	0.18	31	0.0600 ± 0.01^{ab}	25	0.0500 ± 0.02^{a}	20	0.0700 ± 0.02^{b}	**0.005***
FADS1	rs174545	C/G	30	0.0700 ± 0.02	40	0.0600 ± 0.02	17	0.0700 ± 0.03	0.13	31	0.0600 ± 0.02^{ab}	27	0.0600 ± 0.02^{a}	20	0.0800 ± 0.02^{b}	**0.006**
FADS1	rs174546	C/T	30	0.0700 ± 0.02	40	0.0600 ± 0.02	18	0.0700 ± 0.03	0.19	31	0.0600 ± 0.02^{ab}	27	0.0600 ± 0.02^{a}	20	0.0800 ± 0.02^{b}	**0.006**
FADS1	rs174548	C/G	33	0.0600 ± 0.02	40	0.0600 ± 0.02	21	0.0700 ± 0.03	0.21	28	0.0600 ± 0.02^{ab}	27	0.0600 ± 0.02^{a}	17	0.0800 ± 0.02^{b}	**0.005***
FADS1	rs174553	A/G	30	0.0700 ± 0.02	40	0.0600 ± 0.02	18	0.0700 ± 0.03	0.19	31	0.0600 ± 0.02^{ab}	27	0.0600 ± 0.02^{a}	20	0.0800 ± 0.02^{b}	**0.006**
FADS2	rs1535	A/G	30	0.0700 ± 0.02	40	0.0600 ± 0.02	18	0.0700 ± 0.03	0.16	31	0.0600 ± 0.02^{ab}	27	0.0600 ± 0.02^{a}	20	0.0800 ± 0.02^{b}	**0.008**
FADS2	rs174570	C/T	44	0.0700 ± 0.02^{ab}	53	0.0600 ± 0.02^{a}	28	0.0800 ± 0.03^{b}	**0.019**	17	0.0500 ± 0.01^{ab}	13	0.0500 ± 0.02^{a}	10	0.0700 ± 0.01^{b}	**0.020**
FADS2	rs2072114	A/G	47	0.0700 ± 0.02^{ab}	54	0.0600 ± 0.02^{a}	30	0.0800 ± 0.03^{b}	**0.026**	14	0.0600 ± 0.02^{ab}	13	0.0500 ± 0.02^{a}	8	0.0700 ± 0.02^{b}	**0.032**
**AA:LA (D6D+D5D)**																
FADS1	rs174537	G/T	30	0.1600 ± 0.05^{a}	41	0.2000 ± 0.04^{b}	18	0.2200 ± 0.05^{b}	**<0.001***	31	0.1300 ± 0.04^{a}	25	0.1800 ± 0.05^{b}	20	0.2100 ± 0.06^{b}	**<0.001***
FADS1	rs174545	C/G	30	0.1600 ± 0.05^{a}	40	0.2000 ± 0.04^{b}	17	0.2200 ± 0.05^{b}	**<0.001***	31	0.1300 ± 0.04^{a}	27	0.1800 ± 0.05^{b}	20	0.2100 ± 0.06^{b}	**<0.001***
FADS1	rs174546	C/T	30	0.1600 ± 0.05^{a}	40	0.2000 ± 0.04^{b}	18	0.2200 ± 0.05^{b}	**<0.001***	31	0.1300 ± 0.04^{a}	27	0.1800 ± 0.05^{b}	20	0.2100 ± 0.06^{b}	**<0.001***
FADS1	rs174548	C/G	33	0.1500 ± 0.05^{a}	40	0.2000 ± 0.04^{b}	21	0.2200 ± 0.04^{b}	**<0.001***	28	0.1400 ± 0.04^{a}	27	0.1800 ± 0.04^{b}	17	0.2100 ± 0.06^{b}	**<0.001***
FADS1	rs174553	A/G	30	0.1600 ± 0.05^{a}	40	0.2000 ± 0.04^{b}	18	0.2200 ± 0.05^{b}	**<0.001***	31	0.1300 ± 0.04^{a}	27	0.1800 ± 0.05^{b}	20	0.2100 ± 0.06^{b}	**<0.001***
FADS2	rs1535	A/G	30	0.1600 ± 0.05^{a}	40	0.2000 ± 0.04^{b}	18	0.2200 ± 0.05^{b}	**<0.001***	31	0.1300 ± 0.04^{a}	27	0.1800 ± 0.05^{b}	20	0.2100 ± 0.06^{b}	**<0.001***
FADS2	rs174570	C/T	44	0.1600 ± 0.05^{a}	53	0.1900 ± 0.04^{b}	28	0.2200 ± 0.05^{b}	**<0.001***	17	0.1200 ± 0.03^{a}	13	0.1700 ± 0.04^{b}	10	0.1900 ± 0.05^{b}	**<0.001***
FADS2	rs2072114	A/G	47	0.1500 ± 0.05^{a}	54	0.2000 ± 0.04^{b}	30	0.2100 ± 0.05^{b}	**<0.001***	14	0.1300 ± 0.04^{a}	13	0.1600 ± 0.05^{ab}	8	0.2100 ± 0.05^{b}	**0.004***
**AA:DGLA (D5D)**																
FADS1	rs174537	G/T	30	2.5200 ± 0.48^{a}	41	3.6400 ± 0.77^{b}	18	3.6800 ± 2.17^{b}	**<0.001***	31	2.3300 ± 0.81^{a}	25	3.3800 ± 1.02^{b}	20	2.7900 ± 0.71^{b}	**<0.001***
FADS1	rs174545	C/G	30	2.5200 ± 0.48^{a}	40	3.6500 ± 0.79^{b}	17	3.6100 ± 2.21^{a}	**<0.001***	31	2.3300 ± 0.81^{a}	27	3.3800 ± 0.98^{b}	20	2.7900 ± 0.71^{b}	**<0.001***
FADS1	rs174546	C/T	30	2.5200 ± 0.48^{a}	40	3.6500 ± 0.79^{b}	18	3.6800 ± 2.17^{b}	**<0.001***	31	2.3300 ± 0.81^{a}	27	3.3800 ± 0.98^{b}	20	2.7900 ± 0.71^{b}	**<0.001***
FADS1	rs174548	C/G	33	2.5200 ± 0.50^{a}	40	3.6600 ± 0.79^{b}	21	3.6000 ± 2.01^{b}	**<0.001***	28	2.3000 ± 0.83^{a}	27	3.3500 ± 0.97^{b}	17	2.7300 ± 0.75^{ab}	**<0.001***
FADS1	rs174553	A/G	30	2.5200 ± 0.48^{a}	40	3.6500 ± 0.79^{b}	18	3.6800 ± 2.17^{b}	**<0.001***	31	2.3300 ± 0.81^{a}	27	3.3800 ± 0.98^{b}	20	2.7900 ± 0.71^{b}	**<0.001***
FADS2	rs1535	A/G	30	2.5200 ± 0.48^{a}	40	3.6600 ± 0.78^{b}	18	3.6800 ± 2.17^{b}	**<0.001***	31	2.3300 ± 0.81^{a}	27	3.3600 ± 0.98^{b}	20	2.7900 ± 0.71^{b}	**<0.001***
FADS2	rs174570	C/T	44	2.4700 ± 0.72^{a}	53	3.4600 ± 0.83^{b}	28	3.3400 ± 1.85^{b}	**<0.001***	17	2.3000 ± 0.52^{a}	13	3.8800 ± 1.00^{b}	10	2.8500 ± 0.60^{a}	**<0.001***
FADS2	rs2072114	A/G	47	2.4400 ± 0.74^{a}	54	3.5200 ± 0.77^{b}	30	3.2300 ± 1.77^{c}	**<0.001***	14	2.3600 ± 0.38^{a}	13	3.6100 ± 1.25^{b}	8	3.1400 ± 0.91^{ab}	**0.005***
**C18:3n3 (ALA)**																
FADS1	rs174537	G/T	30	0.2100 ± 0.11^{a}	41	0.2300 ± 0.13^{a}	18	0.1400 ± 0.11^{b}	**0.002***	31	0.2500 ± 0.14^{a}	25	0.2500 ± 0.14^{a}	20	0.1500 ± 0.14^{b}	**<0.001***
FADS1	rs174545	C/G	30	0.2100 ± 0.11^{a}	40	0.2400 ± 0.13^{a}	17	0.1300 ± 0.10^{b}	**<0.001***	31	0.2500 ± 0.14^{a}	27	0.2500 ± 0.13^{a}	20	0.1500 ± 0.14^{b}	**<0.001***
FADS1	rs174546	C/T	30	0.2100 ± 0.11^{a}	40	0.2400 ± 0.13^{a}	18	0.1400 ± 0.11^{b}	**0.002***	31	0.2500 ± 0.14^{a}	27	0.2500 ± 0.13^{a}	20	0.1500 ± 0.14^{b}	**<0.001***
FADS1	rs174548	C/G	33	0.2400 ± 0.13^{a}	40	0.2300 ± 0.13^{a}	21	0.1400 ± 0.10^{b}	**<0.001***	28	0.2200 ± 0.12^{a}	27	0.2500 ± 0.14^{a}	17	0.1600 ± 0.15^{b}	**0.002***
FADS1	rs174553	A/G	30	0.2100 ± 0.11^{a}	40	0.2400 ± 0.13^{a}	18	0.1400 ± 0.11^{b}	**0.002***	31	0.2500 ± 0.14^{a}	27	0.2500 ± 0.13^{a}	20	0.1500 ± 0.14^{b}	**<0.001***
FADS2	rs1535	A/G	30	0.2100 ± 0.11^{a}	40	0.2400 ± 0.13^{a}	18	0.1400 ± 0.11^{b}	**0.002***	31	0.2500 ± 0.14^{a}	27	0.2500 ± 0.13^{a}	20	0.1500 ± 0.14^{b}	**<0.001***
FADS2	rs174570	C/T	44	0.2100 ± 0.10^{a}	53	0.2400 ± 0.13^{a}	28	0.1600 ± 0.14^{b}	**<0.001***	17	0.2800 ± 0.17^{a}	13	0.2400 ± 0.14^{a}	10	0.1200 ± 0.06^{b}	**0.002***

FADS2	rs2072114	A/G	47	0.2300 ± 0.12^{a}	54	0.2300 ± 0.13^{a}	30	0.1600 ± 0.14^{b}	**<0.001***	14	0.2200 ± 0.13^{a}	13	0.2700 ± 0.16^{a}	8	0.1000 ± 0.03^{b}	**0.001***
**C20:5n3 (EPA)**																
FADS1	rs174537	G/T	30	0.0900 ± 0.04^{a}	41	0.1400 ± 0.04^{b}	18	0.1200 ± 0.05^{ab}	**<0.001***	31	0.0900 ± 0.06^{a}	25	0.1100 ± 0.03^{b}	20	0.1300 ± 0.08^{b}	**0.009**
FADS1	rs174545	C/G	30	0.0900 ± 0.04^{a}	40	0.1400 ± 0.04^{b}	17	0.1100 ± 0.04^{ab}	**<0.001***	31	0.0900 ± 0.06^{a}	27	0.1100 ± 0.03^{b}	20	0.1300 ± 0.08^{b}	**0.006**
FADS1	rs174546	C/T	30	0.0900 ± 0.04^{a}	40	0.1400 ± 0.04^{b}	18	0.1200 ± 0.05^{ab}	**<0.001***	31	0.0900 ± 0.06^{a}	27	0.1100 ± 0.03^{b}	20	0.1300 ± 0.08^{b}	**0.006**
FADS1	rs174548	C/G	33	0.0900 ± 0.04^{a}	40	0.1400 ± 0.04^{b}	21	0.1200 ± 0.05^{ab}	**<0.001***	28	0.0900 ± 0.06^{a}	27	0.1100 ± 0.03^{b}	17	0.1300 ± 0.08^{b}	**0.011**
FADS1	rs174553	A/G	30	0.0900 ± 0.04^{a}	40	0.1400 ± 0.04^{b}	18	0.1200 ± 0.05^{ab}	**<0.001***	31	0.0900 ± 0.06^{a}	27	0.1100 ± 0.03^{b}	20	0.1300 ± 0.08^{b}	**0.006**
FADS2	rs1535	A/G	30	0.0900 ± 0.04^{a}	40	0.1300 ± 0.04^{b}	18	0.1200 ± 0.05^{ab}	**<0.001***	31	0.0900 ± 0.06^{a}	27	0.1200 ± 0.04^{b}	20	0.1300 ± 0.08^{b}	**0.005***
FADS2	rs174570	C/T	44	0.0900 ± 0.05^{a}	53	0.1300 ± 0.04^{b}	28	0.1400 ± 0.07^{b}	**<0.001***	17	0.0800 ± 0.03	13	0.1100 ± 0.03	10	0.0900 ± 0.03	0.18
FADS2	rs2072114	A/G	47	0.0800 ± 0.04^{a}	54	0.1300 ± 0.04^{b}	30	0.1300 ± 0.07^{b}	**<0.001***	14	0.1100 ± 0.07	13	0.1200 ± 0.04	8	0.1100 ± 0.04	0.51
**C22:5n3 (DPAn3)**																
FADS1	rs174537	G/T	30	0.1800 ± 0.07	41	0.2100 ± 0.12	18	0.1800 ± 0.07	0.57	31	0.2000 ± 0.13	25	0.2200 ± 0.11	20	0.1900 ± 0.07	0.74
FADS1	rs174545	C/G	30	0.1800 ± 0.07	40	0.2100 ± 0.12	17	0.1800 ± 0.07	0.60	31	0.2000 ± 0.13	27	0.2200 ± 0.11	20	0.1900 ± 0.07	0.68
FADS1	rs174546	C/T	30	0.1800 ± 0.07	40	0.2100 ± 0.12	18	0.1800 ± 0.07	0.60	31	0.2000 ± 0.13	27	0.2200 ± 0.11	20	0.1900 ± 0.07	0.67
FADS1	rs174548	C/G	33	0.1800 ± 0.09	40	0.2100 ± 0.12	21	0.1800 ± 0.07	0.43	28	0.2100 ± 0.12	27	0.2100 ± 0.11	17	0.1900 ± 0.07	0.89
FADS1	rs174553	A/G	30	0.1800 ± 0.07	40	0.2100 ± 0.12	18	0.1800 ± 0.07	0.60	31	0.2000 ± 0.13	27	0.2200 ± 0.11	20	0.1900 ± 0.07	0.67
FADS2	rs1535	A/G	30	0.1800 ± 0.07	40	0.2000 ± 0.11	18	0.1800 ± 0.07	0.74	31	0.2000 ± 0.13	27	0.2300 ± 0.12	20	0.1900 ± 0.07	0.48
FADS2	rs174570	C/T	44	0.1900 ± 0.09	53	0.2100 ± 0.12	28	0.1800 ± 0.06	0.65	17	0.1900 ± 0.13	13	0.2100 ± 0.11	10	0.1900 ± 0.08	0.67
FADS2	rs2072114	A/G	47	0.1800 ± 0.10	54	0.2100 ± 0.11	30	0.1800 ± 0.06	0.44	14	0.2200 ± 0.12	13	0.2400 ± 0.12	8	0.2100 ± 0.10	0.91
**C22:6n3 (DHA)**																
FADS1	rs174537	G/T	30	0.5100 ± 0.24^{a}	41	0.9900 ± 0.22^{b}	18	1.2300 ± 0.42^{b}	**<0.001***	31	0.4500 ± 0.25^{a}	25	0.7900 ± 0.27^{b}	20	1.1800 ± 0.46^{c}	**<0.001***
FADS1	rs174545	C/G	30	0.5100 ± 0.24^{a}	40	0.9900 ± 0.22^{b}	17	1.2000 ± 0.43^{b}	**<0.001***	31	0.4500 ± 0.25^{a}	27	0.8200 ± 0.28^{b}	20	1.1800 ± 0.46^{c}	**<0.001***
FADS1	rs174546	C/T	30	0.5100 ± 0.24^{a}	40	0.9900 ± 0.22^{b}	18	1.2300 ± 0.42^{b}	**<0.001***	31	0.4500 ± 0.25^{a}	27	0.8200 ± 0.28^{b}	20	1.1800 ± 0.46^{c}	**<0.001***
FADS1	rs174548	C/G	33	0.4900 ± 0.24^{a}	40	0.9900 ± 0.22^{b}	21	1.2000 ± 0.43^{b}	**<0.001***	28	0.4600 ± 0.26^{a}	27	0.8200 ± 0.29^{b}	17	1.2000 ± 0.45^{c}	**<0.001***
FADS1	rs174553	A/G	30	0.5100 ± 0.24^{a}	40	0.9900 ± 0.22^{b}	18	1.2300 ± 0.42^{b}	**<0.001***	31	0.4500 ± 0.25^{a}	27	0.8200 ± 0.28^{b}	20	1.1800 ± 0.46^{c}	**<0.001***
FADS2	rs1535	A/G	30	0.5100 ± 0.24^{a}	40	0.9900 ± 0.22^{b}	18	1.2300 ± 0.42^{b}	**<0.001***	31	0.4500 ± 0.25^{a}	27	0.8200 ± 0.28^{b}	20	1.1800 ± 0.46^{c}	**<0.001***
FADS2	rs174570	C/T	44	0.5000 ± 0.25^{a}	53	0.9500 ± 0.22^{b}	28	1.3000 ± 0.42^{c}	**<0.001***	17	0.4200 ± 0.21^{a}	13	0.7700 ± 0.33^{b}	10	0.9200 ± 0.35^{b}	**<0.001***
FADS2	rs2072114	A/G	47	0.4700 ± 0.24^{a}	54	0.9600 ± 0.25^{b}	30	1.2300 ± 0.45^{b}	**<0.001***	14	0.5100 ± 0.28^{a}	13	0.7600 ± 0.26^{b}	8	1.0900 ± 0.38^{c}	**<0.001***
**EPA:ALA (D6D+D5D)**																
FADS1	rs174537	G/T	30	0.5400 ± 0.38^{a}	41	0.7500 ± 0.51^{ab}	18	1.1300 ± 0.70^{b}	**0.002***	31	0.5000 ± 0.37^{a}	25	0.5800 ± 0.37^{a}	20	1.0400 ± 0.62^{b}	**<0.001***
FADS1	rs174545	C/G	30	0.5400 ± 0.38^{a}	40	0.7500 ± 0.51^{ab}	17	1.1600 ± 0.71^{b}	**0.002***	31	0.5000 ± 0.37^{a}	27	0.6100 ± 0.37^{a}	20	1.0400 ± 0.62^{b}	**<0.001***
FADS1	rs174546	C/T	30	0.5400 ± 0.38^{a}	40	0.7500 ± 0.51^{ab}	18	1.1300 ± 0.70^{b}	**0.002***	31	0.5000 ± 0.37^{a}	27	0.6100 ± 0.37^{a}	20	1.0400 ± 0.62^{b}	**<0.001***
FADS1	rs174548	C/G	33	0.5000 ± 0.37^{a}	40	0.7600 ± 0.51^{b}	21	1.1500 ± 0.73^{b}	**<0.001***	28	0.5400 ± 0.37^{a}	27	0.5900 ± 0.37^{a}	17	1.0000 ± 0.56^{b}	**0.005***
FADS1	rs174553	A/G	30	0.5400 ± 0.38^{ab}	40	0.7500 ± 0.51^{a}	18	1.1300 ± 0.70^{b}	**0.002***	31	0.5000 ± 0.37^{a}	27	0.6100 ± 0.37^{a}	20	1.0400 ± 0.62^{b}	**<0.001***
FADS2	rs1535	A/G	30	0.5400 ± 0.38^{a}	40	0.7400 ± 0.51^{ab}	18	1.1300 ± 0.70^{b}	**0.002***	31	0.5000 ± 0.37^{a}	27	0.6200 ± 0.37^{a}	20	1.0400 ± 0.62^{b}	**<0.001***

FADS2	rs174570	C/T	44	0.5700 ± 0.40^{a}	53	0.7200 ± 0.50^{a}	28	1.1300 ± 0.66^{b}	**<0.001***	17	0.4000 ± 0.28^{a}	13	0.5700 ± 0.29^{ab}	10	0.9500 ± 0.65^{b}	**0.003***
FADS2	rs2072114	A/G	47	0.4900 ± 0.35^{a}	54	0.7100 ± 0.48^{b}	30	1.0004 ± 0.68^{b}	**<0.001***	14	0.6000 ± 0.45^{a}	13	0.6000 ± 0.40^{a}	8	1.2300 ± 0.53^{b}	**0.011**

Data are means ± standard deviations (SD) of FAs expressed as percentages of the total phospholipid profile. The general linear model and Bonferroni post-hoc test was applied. The analysis was corrected for potential confounders such as pre-gestational IMC, smoking, education and age of mother and infant gender. P-values <0.05 are highlighted in bold and significant differences that persisted after Bonferroni corrections are additionally denoted by stars (*P<0.005). Different superscript letter indicate which groups are different from the others. M: Major allele; m: minor allele; SNP, single nucleotide polymorphism; LA: Linoleic Acid; GLA: gamma-linolenic acid; DGLA: dihomo-gamma-linolenic acid; AA: Arachidonic Acid; AdA: adrenic acid; DPAn6: docosapentaenoic acid n6; ALA: alpha-linolenic Acid; EPA: eicosapentaenoic acid; DPAn3: docosapentaenoic acid n3; DHA: docosahexaenoic Acid.
